# UBE2O, a host ubiquitin-conjugating enzyme, is a key regulator of hepatitis B virus maturation and egress

**DOI:** 10.1016/j.jbc.2025.110750

**Published:** 2025-09-22

**Authors:** Barbora Lubyova, Eva Tikalova, Vaclav Janovec, Boris Ryabchenko, Kristyna Krulova, Vaclav Kropacek, Sandra Huerfano, Ivan Hirsch, Jan Weber

**Affiliations:** 1 Institute of Organic Chemistry and Biochemistry of the Czech Academy of Sciences, Prague, Czech Republic; 2 Department of Genetics and Microbiology, Faculty of Science, Charles University, BIOCEV, Vestec, Czech Republic; 3 Department of Genetics and Microbiology, Faculty of Science, Charles University, Prague, Czech Republic

**Keywords:** hepatitis B virus, HBV core protein, HBV capsid, UBE2O, monoubiquitination, virion maturation, multivesicular bodies, virion egress

## Abstract

A critical step in Hepatitis B virus (HBV) maturation and egress is the ubiquitination of the capsid/core protein (HBc), which enables its recognition by the endosomal sorting complex required for transport (ESCRT) machinery and recruitment to multivesicular bodies (MVBs). This study investigates the role of UBE2O, an atypical E2 ubiquitin-conjugating enzyme with intrinsic E3 ligase activity, in nucleocapsid assembly and virion egress. Loss of UBE2O in HBV-infected primary human hepatocytes (PHH) and HepG2-NTCP cells led to a reduction in viral replication, as evidenced by decreased levels of intracellular HBV DNA, pgRNA, capsids, and extracellular HBeAg. Additionally, UBE2O depletion disrupted intracellular nucleocapsid assembly and impaired the secretion of enveloped virions, but the release of naked nucleocapsids remained unaffected. In contrast, UBE2O overexpression enhanced the secretion of mature virions, whereas the expression of its enzymatically inactive mutant inhibited this process. Additionally, UBE2O mediated the monoubiquitination of hypophosphorylated cytoplasmic HBc and capsids. Subcellular localization experiments using confocal microscopy and proximity ligation assays (PLA) demonstrated that UBE2O colocalizes with capsids and ubiquitinated cargo in CD63-positive MVB compartments, indicating its involvement in the endosomal secretory pathway. Collectively, this study identifies UBE2O and its catalytic activity as key regulators of the HBV virion secretion pathway, highlighting its potential as a therapeutic target for HBV treatment.

Chronic infection with the hepatitis B virus (HBV) is a major public health burden that affects approximately 296 million people worldwide (1, 2). Although the innate immune response to HBV is weak, approximately 90% of infected adults clear the virus completely, presumably through the induction of an effective CD8+ T cell response. In the remaining cases, infection persists and progresses to chronic hepatitis B (CHB), sustained by the stable episomal form of viral DNA, known as covalently closed circular DNA (cccDNA), within hepatocytes. Current therapeutic strategies for CHB, which rely on nucleos(t)ide analogs, effectively suppress viral replication but fail to eradicate the cccDNA reservoir.

The HBV core protein (HBc), also referred to as p21 or HBcAg, plays a central role in the viral life cycle, including replication, nucleocapsid formation, and virion release. HBc comprises 183 or 185 amino acids, depending on the HBV genotype (A to J), and has an approximate molecular weight of 21.5 kDa. This structural protein undergoes posttranslational modifications such as phosphorylation, arginine methylation, ubiquitination, and SUMOylation (3, 4, 5, 6, 7). Among these, dynamic phosphorylation and dephosphorylation of the C-terminal domain (CTD) are particularly important for regulating the multifunctionality of HBc. The functions of HBc include nuclear pore binding and the release of relaxed circular DNA (rcDNA) into the nucleoplasm, pregenomic RNA (pgRNA) encapsidation, nucleocapsid maturation and intracellular trafficking into multivesicular bodies (MVBs) for envelopment and egress (8, 9, 10). While HBc is hyperphosphorylated in empty nucleocapsids, active dephosphorylation is required for pgRNA packaging and rcDNA synthesis (9, 11, 12).

The maturation and release of HBV are complex processes that rely on hijacking host cellular machinery. In particular, HBV utilizes the multivesicular body (MVB) pathway for virion secretion, a process dependent on the endosomal sorting complex required for transport (ESCRT) machinery (13). The ESCRT complex consists of multiple factors (ESCRT-0, -I, -II, -III, and VPS4) that function together to sort and package cargo into intraluminal vesicles (ILVs) within MVBs. A critical step in engaging this pathway is the ubiquitination of the HBV core protein, which is mediated by the NEDD4 family of E3 ubiquitin ligases that recognize a PPAY motif on the HBc protein (14, 15). This ubiquitination acts as a signal, facilitating the recruitment of viral capsids to ESCRT components, such as TSG101, linking the virus to the MVB sorting pathway for its release from the host cell. Thus, NEDD4-dependent ubiquitination of HBc appears to be critical for MVB-mediated HBV egress (15, 16).

Recently, the ubiquitin-conjugating enzyme E2 O (UBE2O) was identified as a potential binding partner of HBc (6). UBE2O is a unique E2/E3 hybrid enzyme that exhibits both E2 ubiquitin-conjugating and E3 ligase activities (17). It primarily targets misfolded or damaged proteins for polyubiquitination and proteasomal degradation. Besides polyubiquitination, UBE2O is also involved in monoubiquitination or multi-monoubiquitination of substrates, such as BAP1 and SMAD6, which regulate their function, subcellular localization, and/or interactions (18, 19, 20). UBE2O has also been implicated in retrograde transport, facilitating endosomal F-actin assembly *via* Lys-63-linked ubiquitination of WASHC1 (21). Furthermore, *UBE2O* is frequently amplified or mutated in multiple cancers, and its high expression is associated with low survival rates of gastric, lung, breast, and prostate cancer patients (17).

Despite UBE2O's established roles in regulating the client protein function, stability, or intracellular trafficking, its potential impact on the HBV life cycle has not been previously investigated. In this study, we examined the interaction of UBE2O with viral HBc and capsids and determined its role in HBV capsid assembly, maturation, and virion egress. Our findings revealed that UBE2O selectively monoubiquitinates hypophosphorylated HBc and capsids. Knockdown of UBE2O in HBV-infected primary human hepatocytes (PHHs) or HepG2-NTCP cells significantly reduced HBV replication, as demonstrated by reduced levels of intracellular HBV DNA, pgRNA, and extracellular HBeAg. Furthermore, UBE2O depletion disrupted hypophosphorylated HBc and nucleocapsid accumulation and virion secretion. We have shown for the first time that UBE2O localizes to CD63- and ubiquitin-positive endosomal compartments, characteristic of MVBs. These findings identify UBE2O as a key regulator of HBV nucleocapsid assembly, trafficking, and virion egress, highlighting its potential as a promising pharmacological target for HBV cure.

## Results

### HBc and capsids interact with UBE2O in both HBV-infected and transfected HepG2-NTCP hepatocytes

**Figure 1:**
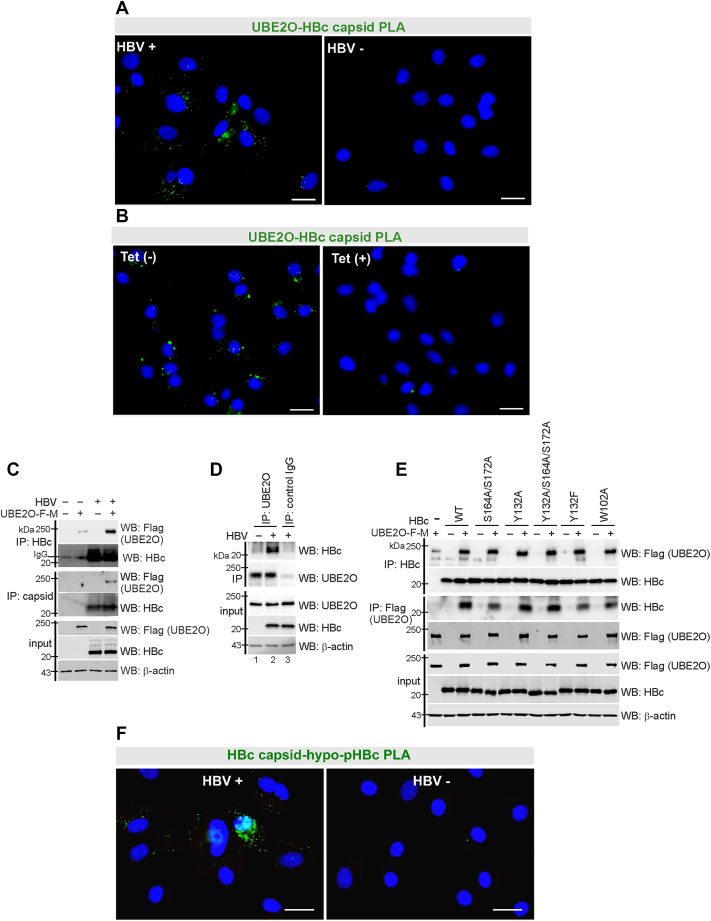
**HBc/capsid interacts with UBE2O.***A*, proximity ligation assay (PLA) using a mouse antibody against the HBV capsid (Hyb-3120) and a rabbit antibody against UBE2O in HBV-infected HepG2-NTCP cells at day 6 post-infection. Nuclei were counterstained with DAPI. Bar, = 20 μm. *B*, PLA using a mouse antibody against the HBV capsid (Hyb-3120) and a rabbit antibody against UBE2O in HepAD38 cells. Tet+, tetracycline-treated cells (low HBV production) and Tet-, tetracycline-untreated cells (high HBV production). Nuclei were counterstained with DAPI. Bar, = 20 μm. *C*, HepG2-NTCP cells were transfected with Flag-Myc-tagged UBE2O and 2 days post-transfection infected with HBV, as indicated. Six days post-infection, protein lysates were immunoprecipitated (IP) using anti-HBc or anti-capsid (Hyb-3120) antibodies and analyzed by Western blotting (WB) with anti-Flag and anti-HBc antibodies. The expression levels of UBE2O and HBc are shown for comparison (input). *D*, HepG2-NTCP cells were infected with HBV, as indicated. Six days post-infection, protein lysates were immunoprecipitated (IP) with anti-UBE2O antibodies (lanes one and 2) or an isotype control antibody (lane 3) and analyzed by Western blotting (WB) with anti-HBc and anti-UBE2O antibodies. The expression levels of endogenous UBE2O, HBc and β-actin are shown for comparison (input). *E*, UBE2O interacts with both capsid assembly-competent (wt, S164A/S172A, and Y132F) and capsid assembly-defective (Y132A, Y132A/S164A/S172A, and W102A) HBc variants. HepG2-NTCP cells were co-transfected with Flag-Myc-tagged UBE2O and wt-HBc or its mutant variants, as indicated. Two days post-transfection, protein lysates were immunoprecipitated (IP) with anti-HBc or anti-Flag antibodies and analyzed by Western blotting (WB) with anti-Flag or anti-HBc antibodies, respectively. The expression levels of UBE2O and HBc are shown for comparison (input). *F*, the PLA assay was performed using a mouse antibody against the HBV capsid (Hyb-3120) and a rabbit antibody against hypo-pHBc in HBV-infected HepG2-NTCP cells at day 6 post-infection. Nuclei were counterstained with DAPI. Bars, = 20 μm.

In our previous study, UBE2O was identified through mass spectrometry as part of a protein complex co-precipitating with HBc in HepG2-NTCP hepatocytes transfected with an HBc expression plasmid (6). In the current study, we validated the interaction between UBE2O and HBc in an infection model. To examine the *in situ* interaction between endogenous UBE2O and HBc during HBV infection, we employed the Duolink proximity ligation assay (PLA) (22, 23), which produces a fluorescent signal when the target proteins are in close proximity (typically <40 nm). Using this approach, we assessed HBc/capsid–UBE2O interactions in HBV-infected HepG2-NTCP cells (Fig. 1*A*) and in HepAD38 cells that stably produce HBV (Fig. 1*B*). For HBV capsid detection, we used the monoclonal antibody Hyb-3120, which specifically recognizes conformational epitopes of HBc capsid (24, 25, 26). PLA signals, which were primarily localized to the cytoplasm (Fig. 1, *A* and *B*), demonstrated spatial proximity between the capsid and UBE2O, supporting the presence of either a direct interaction or an indirect association mediated through a multi-protein complex.

To validate UBE2O–capsid interactions, we performed co-immunoprecipitation and immunoblotting assays (Fig. 1, *C*–*E*). HepG2-NTCP cells were transfected with either a UBE2O-expressing plasmid or an empty vector (pcDNA) and subsequently infected with HBV. At 6 days post-infection, an interaction between HBc/capsids and UBE2O was confirmed (Fig. 1*C*). Similarly, an interaction between endogenous UBE2O and HBc was detected in HBV-infected HepG2-NTCP cells (Fig. 1*D*). Next, we investigated whether UBE2O interacts with HBc in its monomeric or dimeric form or as assembled capsids (Fig. 1*E*). Previous studies have identified HBc amino acid residues Y132 and W102 as critical for capsid assembly (27, 28). Specifically, mutations such as Y132A or W102A disrupt capsid assembly, whereas the substitution of Y132 with phenylalanine (Y132F), which retains a hydrophobic side chain, preserves efficient capsid assembly (Fig. S1*A*). Co-immunoprecipitation assays using plasmids encoding capsid assembly-competent (wild-type, S164A/S172A, and Y132F) and capsid assembly-defective (Y132A and W102A) HBc variants revealed that UBE2O interacts with both HBc monomers/dimers and assembled core particles (Fig. 1*E*). These results indicate that UBE2O associates with HBc irrespective of its assembly state.

HBV nucleocapsid assembly, maturation, and virion egress are associated with rapid HBc protein dephosphorylation (11, 29, 30). We, therefore, determined the subcellular specificity of the UBE2O–HBV capsid interaction detected by the PLA assay, using antibodies against capsids (Hyb-3120) and hypophosphorylated HBc (anti-hypo-pHBc) (Fig. 1*F*). The HBV capsid–hypo-pHBc PLA signal was predominantly detected in the cytoplasm, suggesting that both hypophosphorylated capsids and capsids interacting with UBE2O accumulate in the cytoplasm of infected cells and that they may be targeted for maturation and secretion.

Since this study frequently uses the anti-capsid antibody (Hyb-3120) and three types of anti-HBc-specific antibodies (anti-HBc, anti-hyper-pHBc, and anti-hypo-pHBc), we validated their specificity for capsid conformations (Fig. S1, *B* and *C*) and mapped their epitopes (Fig. S1, *D*–*F*). Particle gel assay (Fig. S1*B*) and immunoprecipitation analyses (Fig. S1*C*) of capsid assembly-competent (wild-type, Y132F, S164A/S172A) and capsid assembly-defective (Y132A and W102A) HBc variants confirmed that the Hyb-3120 antibody specifically binds to capsid-assembled HBc, with a considerably higher affinity for its hypophosphorylated form, S164A/S172A. To determine the HBc epitopes recognized by the three anti-HBc antibodies, we analyzed a series of HBc deletion variants (Fig. S1*D*) along with site-specific amino acid substitutions. The anti-HBc antibody specifically bound to the C-terminal region of HBc (residues 150–185) (Fig. S1*E*). Additionally, the anti-hyper-pHBc antibody selectively recognized phosphorylated serine residues at S164 and S172, while the anti-hypo-pHBc antibody specifically detected the non-phosphorylated epitope spanning S178–S180 (Fig. S1*F*).

### UBE2O is required for HBV replication

**Figure 2:**
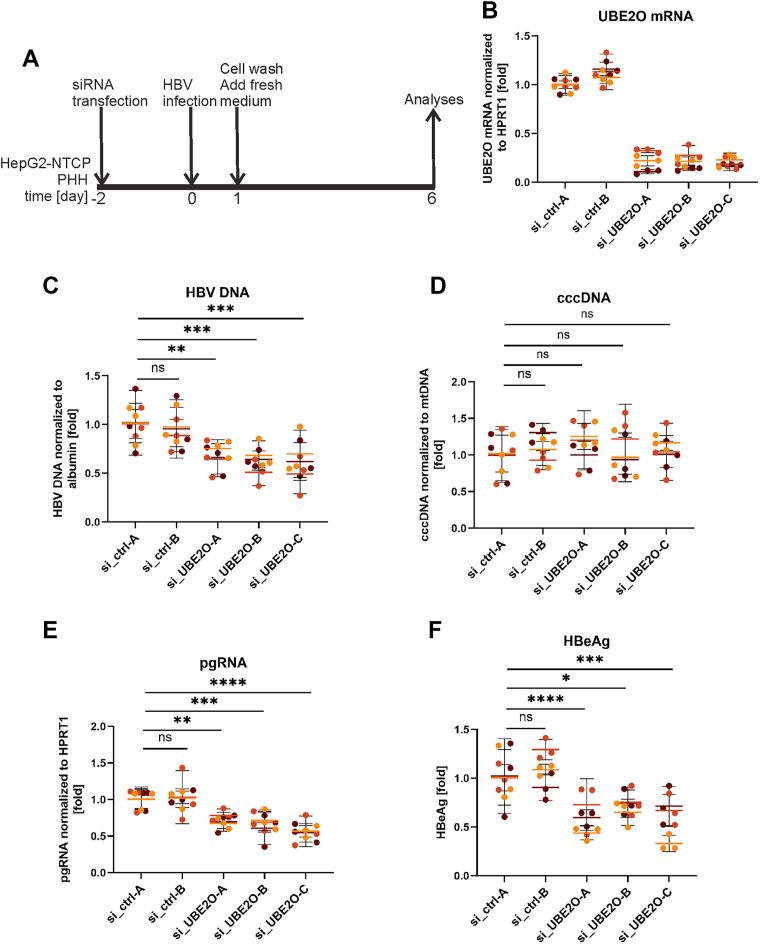
**Inhibition of UBE2O expression reduces HBV replication and secretion of HBe antigen in infected HepG2-NTCP cells**. *A*, experimental outline: HepG2-NTCP cells or primary human hepatocytes (PHH; in Fig. S2) were transfected with control (si_ctrl-A, si_ctrl-B) or UBE2O-specific (si_UBE2O-A, si_UBE2O-B and si_UBE2O-C) siRNAs. Two days after transfection, the cells were infected with the HBV. Six days post-infection, the cells and media were harvested to quantify the expression levels of *UBE2O* (in *B*), intracellular total HBV DNA (in *C*), cccDNA (in *D*), pgRNA (in *E*), and the extracellular HBe antigen (in *F*). Data are presented as the mean ± SD from three independent experiments. Color-coded data points distinguish individual replicates from each experiment, and colored horizontal lines indicate the mean values. Statistical significance of differences between UBE2O silencing and control (si_ctrl-A) groups was evaluated by 2-way ANOVA (*p* ≥ 0.05 – not significant (ns); ∗*p* < 0.05; ∗∗*p* < 0.01; ∗∗∗*p* < 0.001; ∗∗∗∗*p* < 0.0001).

To address the potential role of UBE2O in the HBV life cycle, we used UBE2O-specific siRNAs and analyzed the replication of the virus in HBV-infected HepG2-NTCP cells (Fig. 2) and primary human hepatocytes (PHH) (Fig. S2) using the same experimental layout (Fig. 2*A*). In experiments with HepG2-NTCP cells, three UBE2O-specific siRNAs (UBE2O-A, -B, -C) were used to downregulate UBE2O. However, due to the limited availability of PHHs, all experiments in PHHs were performed using only two UBE2O-specific siRNAs (si_UBE2O-B and -C). The specificity, knockdown efficiency, and potential cytotoxicity of all siRNAs used (both UBE2O-specific and non-targeting controls) were tested using RT-qPCR (Figs. 2*B* and S2*A*), Western blotting and XTT assays (Fig. S3, *A* and *B*). HBV replication was assessed by measuring intracellular viral DNA, cccDNA, pregenomic RNA (pgRNA), and HBe antigen secretion. UBE2O downregulation in HepG2-NTCP cells led to an approximate 50% reduction in all viral parameters, except for cccDNA (Fig. 2, *C*–*F*). A similar decrease in HBV replication parameters was also observed in HBV-infected PHH, with cccDNA levels remaining unchanged (Fig. S2, *B*–*E*). These findings highlight UBE2O's significant role in supporting HBV replication.

### Multiple effects of UBE2O on morphogenesis of virus particle: UBE2O silencing impairs nucleocapsid formation and reduces hypophosphorylated core protein level

**Figure 3:**
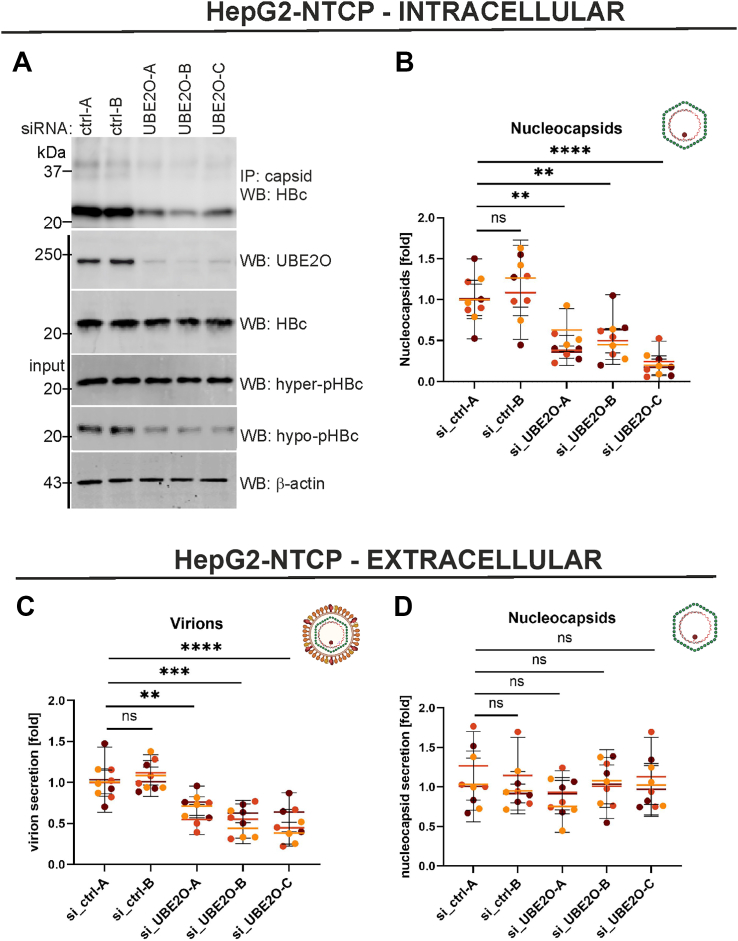
**UBE2O knockdown in HBV-infected HepG2-NTCP cells led to the inhibition of intracellular nucleocapsid assembly and the secretion of enveloped virions**. HepG2-NTCP cells were transfected with two control and three UBE2O-specific siRNAs. Two days post-transfection, the cells were infected with the HBV according to the experimental outline shown in Figure 2*A*. Six days post-infection, the cells and media were harvested for analyses of nucleocapsid assembly and virion secretion. *A*, the protein extracts isolated from HBV-infected HepG2-NTCP cells were analyzed by Western blotting (WB; input) and subjected to immunoprecipitation with anti-capsid (Hyb-3120) antibodies (IP: capsid, WB: HBc). *B*, downregulation of UBE2O expression in infected HepG2-NTCP cells resulted in decreased levels of intracellular DNA-containing nucleocapsids. Nucleocapsids were immunoprecipitated from protein lysates using anti-capsid antibodies and quantified *via* qPCR. *C*, Downregulation of UBE2O expression in infected HepG2-NTCP cells resulted in decreased levels of secreted enveloped virions. Viral particles were immunoprecipitated from the cell culture supernatants using anti-S antibodies and quantified *via* qPCR. *D*, the secretion of naked nucleocapsids was not affected by UBE2O downregulation. Naked nucleocapsids were immunoprecipitated from the cell culture supernatants using anti-capsid antibodies, followed by quantification *via* qPCR. In (B-D), data are presented as the mean ± SD from three independent experiments. Color-coded data points distinguish individual replicates from each experiment, and colored horizontal lines indicate the mean values. The statistical significance of differences between UBE2O silencing and control (si_ctrl-A) groups was evaluated by 2-way ANOVA (*p* ≥ 0.05 – not significant (ns); ∗∗*p* < 0.01; ∗∗∗*p* < 0.001; ∗∗∗∗*p* < 0.0001).

Next, we evaluated the intracellular levels of core protein and viral nucleocapsids in HBV-infected HepG2-NTCP cells (Fig. 3) and PHH (Fig. S4). While UBE2O knockdown caused a slight reduction in total and hyperphosphorylated HBc levels (Figs. 3*A* and S4*A*; WB: HBc and hyper-pHBc), the levels of hypophosphorylated HBc and capsids were significantly decreased in both cell models (Figs. 3*A* and S4*A*; WB: hypo-pHBc; IP: capsid).

The HBV capsid pool comprises both empty capsids and those containing viral RNA or DNA (12). To quantify DNA-associated nucleocapsids, we performed immunoprecipitation of capsids using anti-capsid antibodies, followed by DNA analysis through qPCR. Knockdown of UBE2O led to a significant reduction of viral nucleocapsids in both HepG2-NTCP cells (Fig. 3*B*) and PHH (Fig. S4*B*), indicating an essential role of UBE2O in facilitating HBV nucleocapsid formation.

### UBE2O silencing inhibits the secretion of enveloped virions but not naked nucleocapsids

HBV-replicating cells release both infectious enveloped particles and naked capsids lacking the HBs envelope (31, 32). While virion secretion relies on multivesicular bodies (MVBs) and the ESCRT machinery, naked capsids are released *via* an ESCRT-independent pathway involving the Alix protein (33, 34). To distinguish between these two HBV particle types, media from infected HepG2-NTCP cells or PHH were precipitated with anti-S or anti-capsid antibodies, followed by DNA purification and qPCR analysis. UBE2O knockdown significantly reduced the secretion of enveloped virions (Figs. 3*C* and S4*C*) but did not affect the release of naked nucleocapsids (Figs. 3*D* and S4*D*). These results suggest that UBE2O is specifically involved in the pathway required for virion secretion.

### UBE2O and viral capsids co-localize within CD63-positive multivesicular bodies (MVBs)

**Figure 4:**
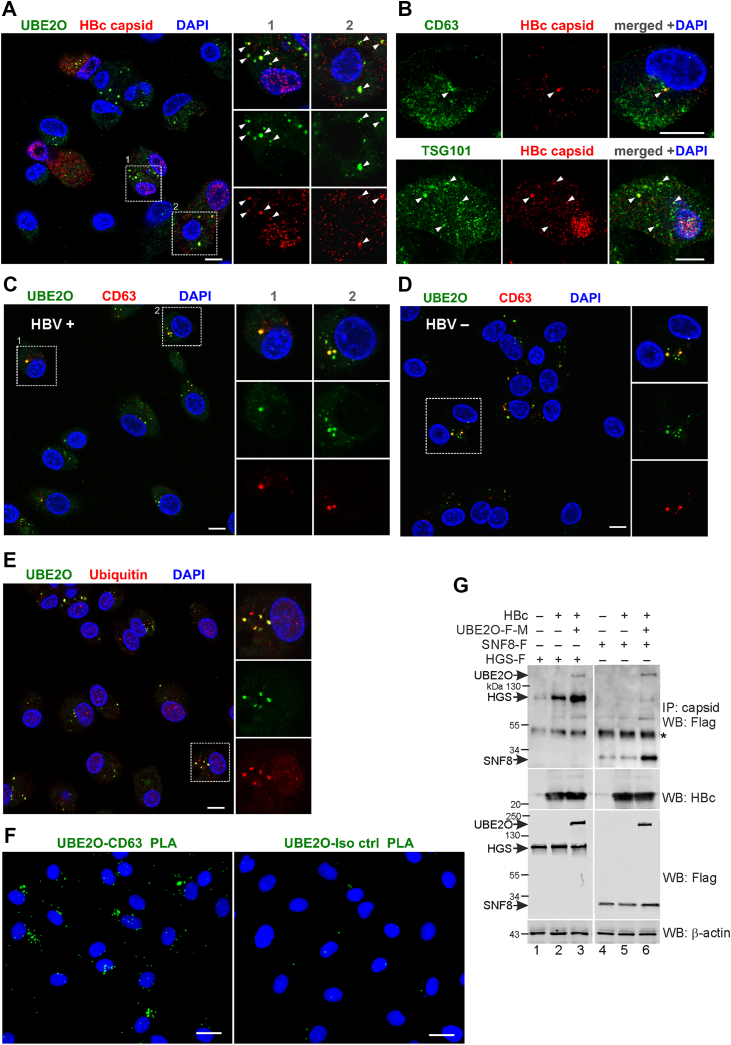
**UBE2O and viral capsids associate with MVBs in HBV-infected HepG2-NTCP cells.***A–F*, HepG2-NTCP cells were either infected with HBV or maintained as uninfected controls. Six days post-infection, cells were immunostained for confocal microscopy analysis (in *A–E*) or subjected to PLA (in *F*). *A*, HBV-infected HepG2-NTCP cells were immunostained with anti-capsid (*red*) and anti-UBE2O (*green*) antibodies. White squares indicate enlarged areas of UBE2O and HBV capsid co-localization shown on the *right*. Nuclei were counterstained with DAPI. Bar, = 10 μm. *B*, colocalization of HBV capsids (*red*) with CD63 or TSG101 (both *green*) in HBV-infected HepG2-NTCP cells. DAPI was used for nuclear staining. Bars, = 10 μm. *C*, HBV-infected or (*D*) uninfected HepG2-NTCP cells were stained with antibodies against UBE2O (*green*) and CD63 (*red*). *White* squares indicate enlarged regions of UBE2O and CD63 co-localization, as shown on the *right*. Nuclei were counterstained with DAPI. Bars, = 10 μm. *E*, confocal section of HBV-infected HepG2-NTCP cells. Ubiquitin (*red*) and UBE2O (*green*) were stained with specific antibodies, and nuclei were stained with DAPI. *White* squares indicate enlarged areas of UBE2O and ubiquitinated proteins co-localization, shown on the *right*. Bars, = 10 μm. *F*, PLA of the interaction between UBE2O and CD63 in HBV-infected HepG2-NTCP cells was measured 6 days post-infection using Duolink PLA reagents. A mouse isotype antibody was used as a control. Nuclei were counterstained with DAPI. Bars, = 20 μm. *G*, HepG2-NTCP cells were transfected with wt-HBc, Flag-Myc-tagged wt-UBE2O, Flag-tagged SNF8/EAP30, or Flag-tagged HGS, as indicated. Isolated protein lysates were subjected to immunoprecipitation with anti-capsid antibodies followed by Western blotting with anti-Flag antibodies to detect HGS, SNF8 and UBE2O. The expression levels of transfected proteins were analyzed by Western blotting (*bottom panels*; WB: Flag and WB: HBc). ∗, IgG heavy chain.

To investigate the subcellular localization of UBE2O and its association with HBV capsids, we performed confocal microscopy on HBV-infected HepG2-NTCP cells (Fig. 4). While UBE2O formed cytoplasmic clusters with minimal nuclear localization, viral capsids were distributed across the cytoplasm and nucleus. Interestingly, the cytoplasmic capsids prominently accumulated within UBE2O-positive clusters (Fig. 4*A*).

Given the established roles of MVBs and autophagic compartments in nucleocapsid maturation and virion assembly (15, 31, 35, 36, 37), we co-stained capsids and MVB markers, CD63 and TSG101. As shown in Figure 4*B*, HBV capsids formed large cytoplasmic aggregates co-localizing with these markers. Because our data also suggest that UBE2O is important for nucleocapsid assembly and virion egress, we examined the association of UBE2O with CD63-positive MVBs in HBV-infected cells. As shown in Figure 4*C*, UBE2O is extensively co-localized with CD63 clusters, indicating its connection to MVBs. To further characterize the structure of CD63 compartments, we performed Z-stack imaging of CD63 and UBE2O using confocal microscopy (Video S1). UBE2O accumulated both within large CD63-positive spherical clusters and outside these clusters, suggesting its dynamic shuttling between MVBs and other cytoplasmic compartments. Importantly, the co-localization of UBE2O and CD63 was not influenced by HBV infection, as this interaction was also observed in non-infected cells (Fig. 4*D*). Immunofluorescence analyses of UBE2O (Fig. S5*A*) and CD63 (Fig. S5*B*), in conjunction with cellular membrane staining using Abberior STAR ORANGE membrane probe, demonstrated that both proteins associate with intracellular membranes within the cytoplasm, further supporting their involvement in multivesicular body (MVB) cargo formation and sorting. Using an antibody specific for mono- and polyubiquitinated proteins, we confirmed the accumulation of ubiquitinated cargo in UBE2O-positive clusters (Fig. 4*E*). PLA further validated the close association between UBE2O and CD63 (Fig. 4*F*), linking UBE2O to the MVB endosomal pathway.

We also examined the interaction of capsids with ESCRT-0 and ESCRT-II components, HGS and SNF8/EAP30. Capsids associated with HGS independently of UBE2O but showed enhanced interaction following UBE2O overexpression (Fig. 4*G*, lanes 2 and 3). In contrast, capsid–SNF8/EAP30 association occurred only in the presence of UBE2O (Fig. 4*G*, lane 6).

Collectively, our data indicate that UBE2O localizes to MVBs and may participate in the endosomal sorting of viral capsid cargo.

### UBE2O monoubiquitinates hypophosphorylated HBc and capsids

**Figure 5:**
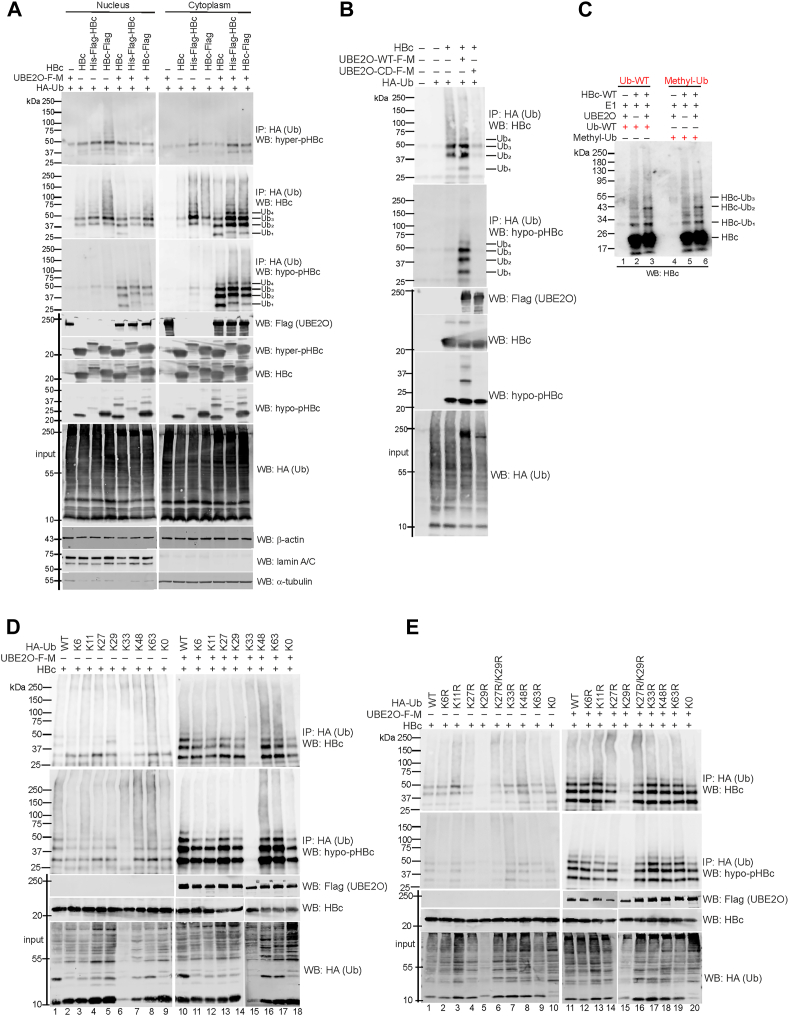
**UBE2O mediates the monoubiquitination of hypophosphorylated HBc localized in the cytoplasm**. *A*, HepG2-NTCP cells were transfected with HA-tagged ubiquitin (HA-Ub) together with Flag-Myc-tagged wt-UBE2O and various wt-HBc expression plasmids that were either untagged (HBc) or tagged at the N-terminus (His-Flag-HBc) or the C-terminus (HBc-Flag). Forty-eight hours after transfection, the cells were fractionated into nuclear (*left*) and cytoplasmic (*right*) extracts. The protein lysates were denatured in 2% SDS and analyzed by immunoprecipitation (IP: anti-HA) followed by Western blotting (WB: hyper-pHBc, HBc and hypo-pHBc). The expression levels of HBc, UBE2O, ubiquitin, lamin A/C (nuclear marker), α-tubulin (cytoplasmic marker) and β-actin (loading control) were analyzed in the input samples. *B*, the catalytically-defective mutant UBE2O-CD (UBE2O-C1040S) lost the ability to ubiquitinate the HBc/capsids. HepG2-NTCP cells were transfected with HA-tagged ubiquitin, wt-HBc and Flag-Myc-tagged UBE2O-wt or UBE2O-CD. The whole cell lysates were isolated, denatured and analyzed as in (*A*). *C*, *in vitro* ubiquitination of wt-HBc with 400 nM UBE2O, 75 nM E1 enzyme, 5 μM ubiquitin-wt (Ub-WT) or methylated ubiquitin (Methyl-Ub). Western blotting with anti-HBc antibodies was used to visualize ubiquitinated HBc, representative of two replicates. *D and E*, immunoprecipitation and Western blot analysis of cellular lysates from HepG2-NTCP cells transfected with wt-HBc, Flag-Myc-tagged wt-UBE2O and HA-tagged single K (in *D*) or K-to-R (in *E*) ubiquitin mutants. The ubiquitinated proteins were precipitated with anti-HA antibodies (IP: HA) and analyzed by Western blotting with anti-HBc or anti-hypo-pHBc.

HBc ubiquitination is believed to be important for capsid trafficking to MVBs and virion secretion (16). To investigate whether UBE2O ubiquitinates HBc and/or capsids, HepG2-NTCP cells were co-transfected with HBc-, UBE2O-, and HA-ubiquitin-expressing plasmids. Western blot analyses of anti-HA immunoprecipitated complexes revealed that UBE2O preferentially monoubiquitinates cytoplasmic hypophosphorylated HBc. Four major bands for HBc were detected (Fig. 5*A*), migrating at approximate molecular weights of 30, 39, 48 and 57 kDa. These molecular weight shifts corresponded to covalent modifications of HBc by one to four ubiquitin moieties. Importantly, the HBc monoubiquitination was not detected when the cells were co-transfected with catalytically defective UBE2O (C1040S, UBE2O-CD) (Fig. 5,*B*).

To confirm that UBE2O mediates HBc monoubiquitination, we performed an *in vitro* ubiquitination assay using recombinant active UBE2O and *in vitro* translated HBc-wt (Fig. 5*C*). The use of chain-terminating methylated ubiquitin (Methyl-Ub) allowed for the specific assessment of mono- and multi-monoubiquitination events. To minimize the contribution of endogenous ubiquitin present in the rabbit reticulocyte lysate (RRL) used for HBc synthesis, the lysate was diluted 25-fold (1 μl of *in vitro* translated HBc in a 25 μl total reaction), and an excess of either wild-type ubiquitin (Ub-wt, 5 μM) or chain-terminating methylated ubiquitin (Methyl-Ub, 5 μM) was supplied. Upon addition of UBE2O, the reaction yielded two prominent bands corresponding to HBc-Ub_1_ and HBc-Ub_2_, along with a faint, less intense band representing HBc-Ub_3_ (Fig. 5*C*, lanes three and 6). The pattern and intensity of HBc-ubiquitin conjugates were similar in reactions with either wild-type or methylated ubiquitin. In conclusion, *in vitro* data demonstrate that UBE2O catalyzes the attachment of up to three ubiquitin moieties to HBc, indicating its capacity for direct mono- and multi-monoubiquitination. This observation is further supported by cellular experiments using various Ub-K and Ub-K/R mutants, as well as the ubiquitin K0 variant, a lysine-less form of ubiquitin in which all seven lysine residues are mutated to arginine, making it incapable of forming polyubiquitin chains (Fig. 5, *D* and *E*). Co-transfection with the HA-Ub-K0 variant similarly limited HBc modification to a maximum of three ubiquitin conjugates (Fig. 5*D*, lane 18; Fig. 5*E*, lane 20), consistent with findings from *in vitro* experiments and confirming the role of UBE2O in mediating HBc mono- and multi-monoubiquitination. We note that the K33 and K29R ubiquitin mutants accumulated only at very low levels in cells, which likely explains the lack of detectable HBc modification in these lanes.

**Figure 6:**
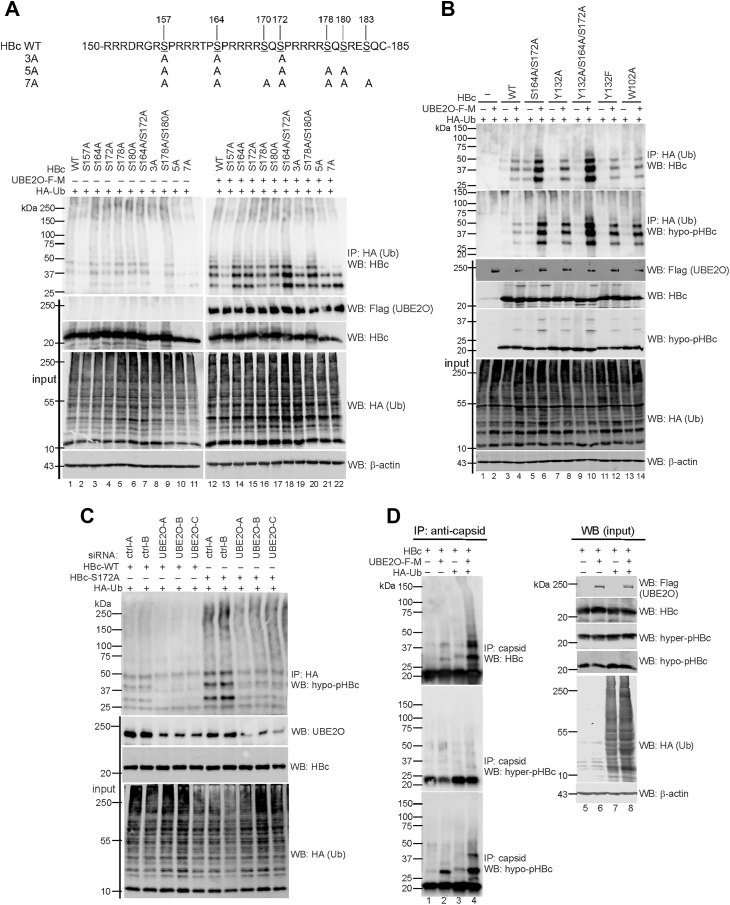
**Inhibition of HBc/capsid phosphorylation at S164 and S172 enhances UBE2O-mediated monoubiquitination of HBc/capsid**. *A*, *top*–Schematic representation of serine-to-alanine mutations in the CTD of HBc. The sequence of wild-type HBc corresponds to HBV genotype A, subtype adw2. *Bottom* – Analysis of UBE2O-mediated ubiquitination of single or multiple S-to-A HBc mutants. The images display analysis of denatured whole cell lysates immunoprecipitated with anti-HA antibodies (IP: anti-HA) and analyzed by Western blotting with anti-HBc antibodies. *B*, analysis of UBE2O-mediated ubiquitination of both capsid assembly-competent (wt, S164A/S172A, and Y132F) and capsid assembly-defective (Y132A, Y132A/S164A/S172A, and W102A) HBc mutants. The images display analyses of denatured whole cell lysates immunoprecipitated with anti-HA antibodies (IP: anti-HA) and analyzed by Western blotting with anti-HBc and anti-hypo-pHBc antibodies. *C*, downregulation of UBE2O led to reduced HBc ubiquitination. HepG2-NTCP cells were transfected with control and UBE2O-specific siRNAs together with HA-Ub, wt-HBc, or S172A-HBc mutant. Denatured protein lysates were immunoprecipitated with anti-HA (Ub) antibodies and precipitated complexes were analyzed by Western blotting with anti-hypo-pHBc antibodies. The expression levels of UBE2O, HBc and ubiquitin were analyzed in the input samples. *D*, monoubiquitinated HBc assembles into capsids. HepG2-NTCP cells were transfected with wt-HBc, Flag-Myc-tagged wt-UBE2O and HA-tagged ubiquitin, as indicated. The cytoplasmic lysates were subjected to immunoprecipitation with anti-capsid antibodies (IP: anti-capsid, panels on the left, lanes one–4), followed by Western blotting with anti-HBc, anti-hyper-pHBc and anti-hypo-pHBc. The expression levels of HBc, ubiquitin and UBE2O were analyzed by Western blotting (input, *panels* on the *right*, lanes five–8).

Detailed mapping studies identified serines in the HBc CTD as crucial residues for ubiquitination (Fig. 6*A*). Mutation S157A reduced ubiquitination, while single or double mutations at S164 and S172 enhanced it (Fig. 6*A*), suggesting that hypophosphorylation of S164 and S172 is essential for HBc monoubiquitination mediated by UBE2O.

UBE2O was shown to monoubiquitinate both capsid assembly-competent (WT, Y132F) and capsid assembly-defective (Y132A and W102A) HBc variants (Fig. 6*B*). Notably, the hypophosphorylated forms of capsids (HBc-S164A/S172A) or HBc monomers/dimers (HBc-Y132A/S164A/S172A) exhibited the highest levels of ubiquitination (Fig. 6*B*, lanes 6 and 10). In further agreement with our results, in UBE2O-deficient cells, the ubiquitination of wt-HBc and the S172A mutant was significantly reduced (Fig. 6*C*). Finally, we determined whether monoubiquitinated HBc protein could be assembled into capsids (Fig. 6*D*). Protein lysates from HepG2-NTCP cells transfected with wt-HBc, wt-UBE2O, and ubiquitin (Ub) expression plasmids were immunoprecipitated with anti-capsid antibodies and analyzed by Western blotting with anti-HBc, -hyper-pHBc, and -hypo-pHBc antibodies. While the expression of UBE2O was associated with a moderate increase of monoubiquitinated capsids (Fig. 6*D*, lane 2), the co-expression of UBE2O and ubiquitin led to a marked increase in monoubiquitinated capsid structures (Fig. 6*D*, lane 4).

### UBE2O regulates HBc/capsid trafficking and secretion

**Figure 7:**
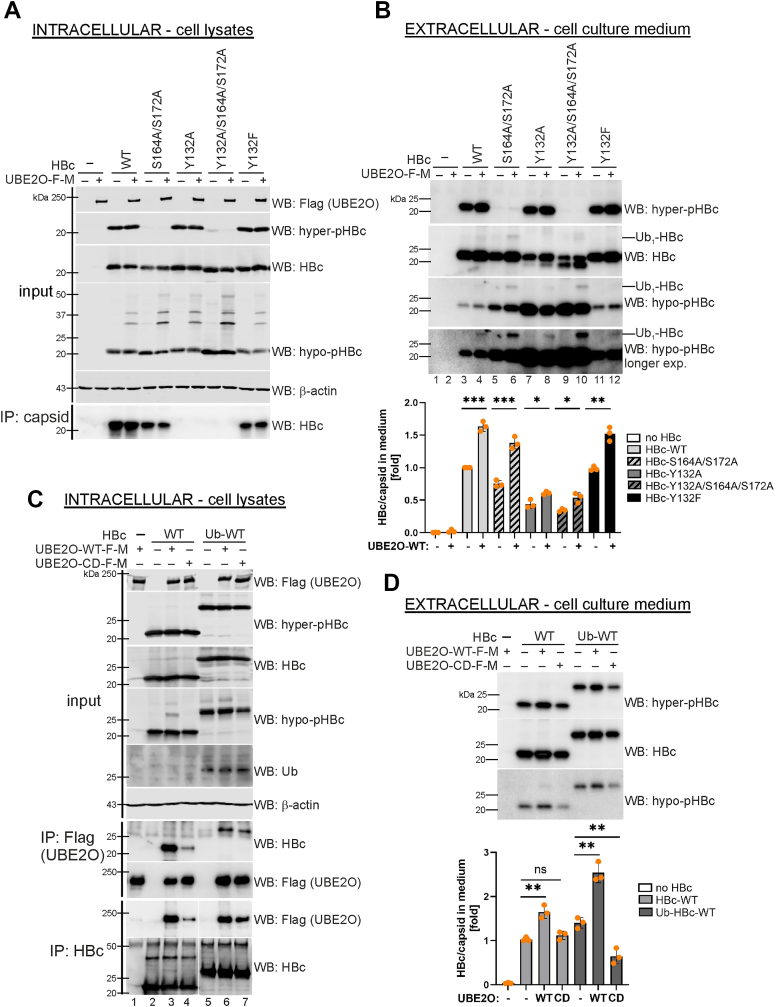
**UBE2O stimulates HBc/capsid secretion**. *A* and *B*, analysis of capsid assembly-competent (wt, S164A/S172A, and Y132F) and capsid assembly-defective (Y132A and Y132A/S164A/S172A) HBc variants’ secretion. HepG2-NTCP cells were co-transfected with UBE2O-wt and HBc variants, as indicated. *A*, the expression levels of intracellular UBE2O and HBc in protein lysates were determined by Western blotting (WB; input). The ability of HBc variants to assemble into capsids was examined by immunoprecipitation with anti-capsid (Hyb-3120) antibodies (IP: capsid; WB: HBc). *B*, the levels of secreted HBc were analyzed in the cell culture supernatants of transfected cells by Western blotting with HBc, hyper-pHBc and hypo-pHBc antibodies (Extracellular). Graph – Quantification of secreted HBc levels was performed by densitometric analysis of Western blots probed with anti-HBc antibodies, using ImageQuant TL Array software. The data are presented as means ± SDs of one experiment performed in biological replicates (n = 3) shown by orange points. The statistical significance of differences between the control and UBE2O-wt or UBE2O-CD groups was evaluated by a two-tailed *t* test (∗*p* < 0.05; ∗∗*p* < 0.01; ∗∗∗*p* < 0.001). *C and D*, effect of UBE2O-wt and catalytically-defective (CD) mutant on the secretion of HBc and Ub-fused HBc. *C*, HepG2-NTCP cells were co-transfected with UBE2O-wt or -CD together with HBc (WT) and Ub-fused HBc (Ub-WT) variant, as indicated. The isolated cytoplasmic protein extracts (Intracellular) were analyzed by immunoprecipitation with anti-Flag (IP:Flag) or anti-HBc (IP:HBc) antibodies followed by Western blotting with anti-HBc or anti-Flag, respectively. The expression levels of UBE2O, HBc, and β-actin (loading control) were analyzed in the input samples (input). *D*, the levels of secreted HBc and Ub-HBc were analyzed in the medium of transfected cells by Western blotting with anti-HBc, anti-hyper-pHBc and anti-hypo-pHBc antibodies (Extracellular). Graph–Quantitative analysis of secreted HBc and Ub-HBc determined by ELISA in the cell culture supernatants. The data are presented as means ± SDs of one experiment performed in biological replicates (n = 3) shown by orange points. The statistical significance of differences between the control and UBE2O-wt or UBE2O-CD groups was evaluated by a two-tailed *t* test (*p* ≥ 0.05–not significant (ns); ∗∗*p* < 0.01).

Considering UBE2O’s implication in virion egress, we examined its role in modulating the trafficking and secretion of HBc and empty capsid particles in transfected HepG2-NTCP cells. Cell lysates (Fig. 7*A*) and cell culture supernatants (Fig. 7*B*) from cells transfected with UBE2O and either capsid assembly-competent or -defective variants were analyzed by immunoblot. The ability of HBc variants to assemble into capsids was determined by immunoprecipitation (Fig. 7*A*; IP: capsid). The overexpression of UBE2O significantly enhanced the secretion of all HBc variants (Fig. 7*B*). Notably, cells expressing capsid assembly-defective HBc variants (Y132A and Y132A/S164A/S172A) secreted high levels of hypophosphorylated HBc (Fig. 7*B*, lanes 7 and 9). While UBE2O overexpression partially inhibited this constitutive secretion, it also facilitated the release of hyperphosphorylated HBc and the monoubiquitinated form of hypophosphorylated HBc (Fig. 7*B*, Lanes 8 and 10). Since capsid assembly-defective HBc variants can only form dimers, it is reasonable to suggest that hypophosphorylated HBc monomers or dimers are constitutively secreted, likely through the ER-Golgi pathway, following the same secretory route as HBeAg. In contrast, UBE2O overexpression may interfere with this constitutive pathway and promote HBc secretion *via* the MVB-mediated secretory pathway.

### HBc monoubiquitination regulates its subcellular localization and interaction with UBE2O

UBE2O is a large, multifunctional ubiquitin enzyme composed of 1292 amino acids. It contains three conserved regions (CR1–CR3), a predicted coiled-coil (CC) domain, and a catalytic ubiquitin-conjugating (UBC) domain (Fig. S6*A*). Its enzymatic activity depends on a conserved cysteine residue (C1040) within the UBC domain, which is essential for forming a thioester bond with ubiquitin during substrate modification. Mutation of this catalytic cysteine (C1040S) not only abolishes enzymatic activity but also disrupts substrate binding, likely due to conformational changes in the UBC domain that destabilize the substrate-binding interface (38).

Structural and mechanistic studies by Yip *et al.* (38) revealed that attachment of a single ubiquitin moiety to a substrate can enhance UBE2O binding and promote further multi-monoubiquitination. Through this feed-forward mechanism, UBE2O favors mono- and multi-monoubiquitination over the formation of conventional polyubiquitin chains.

To test whether HBc monoubiquitination facilitates UBE2O binding and triggers its multi-monoubiquitination, we generated a ubiquitin-fused HBc variant (Ub-HBc), in which ubiquitin harboring a G76V mutation (to prevent cleavage by deubiquitinases) was fused to the N-terminus of HBc (Fig. S6*B*). Immunoprecipitation analyses demonstrated that both the wild-type HBc (HBc-wt) and its ubiquitin-fused variant (Ub-HBc-wt) were capable of assembling into capsids (Fig. S6*C*, IP: capsid). Nevertheless, their subcellular localization exhibited marked differences: while HBc-wt was distributed across both the nucleus and cytoplasm, Ub-HBc-wt was predominantly confined to the cytoplasm, with no detectable nuclear translocation (Fig. S6*C*). Despite these distinct intracellular localization patterns, both variants were effectively secreted into the extracellular cell culture supernatants (Fig. S6*D*).

Co-immunoprecipitation experiments confirmed the interaction of wild-type HBc (HBc-wt) with wild-type UBE2O (UBE2O-wt) but not with the catalytically-defective (CD) UBE2O mutant (Fig. 7*C*, IP: Flag, IP: HBc, lanes 3, 4). In contrast, the Ub-fused HBc enabled interaction with both UBE2O-wt and the CD mutant (Fig. 7*C*, IP: Flag, IP: HBc, lanes 6, 7), suggesting that the association between UBE2O and HBc may also be mediated through ubiquitin binding. Analysis of the cell supernatants revealed that while UBE2O-wt markedly enhanced the secretion of both HBc and Ub-HBc, the catalytically inactive UBE2O-CD mutant inhibited the secretion of Ub-HBc (Fig. 7*D*, Extracellular). These findings demonstrate the critical importance of UBE2O's E2/E3 enzymatic activity in regulating the release of HBc/capsid.

### UBE2O enhances multi-monoubiquitination of HBc/capsids and promotes their secretion

**Figure 8:**
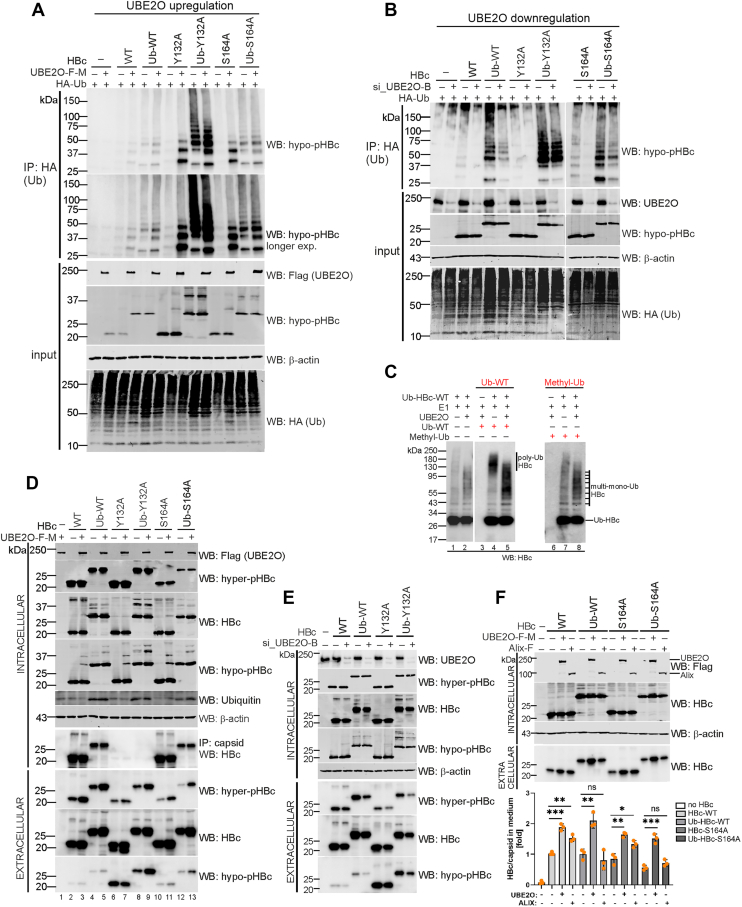
**UBE2O mediates HBc/capsid multi-monoubiquitination and secretion.***A*, UBE2O stimulates HBc and capsid monoubiquitination and multi-monoubiquitination. HepG2-NTCP cells were transfected with Flag-Myc-tagged UBE2O-wt (UBE2O upregulation) together with HA-tagged ubiquitin and various HBc (WT, Y132A, and S164A) or Ub-HBc (Ub-WT, Ub-Y132A, and Ub-S164A) variants. The denatured whole cell lysates were immunoprecipitated with anti-HA antibodies (IP: anti-HA) and analyzed by Western blotting with anti-hypo-pHBc antibodies. The expression levels of UBE2O, HBc, and β-actin (loading control) were analyzed in the input samples (input). *B*, UBE2O depletion leads to the reduction of HBc and capsid monoubiquitination and multi-monoubiquitination. HepG2-NTCP cells were transfected with UBE2O-specific siRNA (si_UBE2O-B; UBE2O downregulation) together with HA-tagged ubiquitin and various HBc or Ub-HBc variants. The samples were analyzed as in (*A*). *C*, *in vitro* ubiquitination of Ub-HBc-wt with 400 nM UBE2O, 75 nM E1 enzyme, 5 μM ubiquitin-wt or methylated ubiquitin, as indicated. Western blotting with anti-HBc antibodies was used to visualize ubiquitinated Ub-HBc, representative of two replicates. *D* and *E*, Effect of UBE2O overexpression (in *D*) or downregulation (in *E*) on the secretion of various HBc and Ub-HBc variants. HepG2-NTCP cells were co-transfected with UBE2O-wt (*D*) or UBE2O-specific siRNA (si_UBE2O-B, (E)) together with various HBc and Ub-HBc variants, as indicated. The expression levels of UBE2O, HBc, and β-actin (loading control) were analyzed in the input samples (intracellular). The ability of HBc variants to assemble into capsids was determined by immunoprecipitation with anti-capsid (Hyb-3120) antibodies (IP: capsid; WB: HBc). The levels of secreted HBc and Ub-HBc were analyzed in the cell culture supernatants of transfected cells by Western blotting with anti-HBc, anti-hyper-pHBc and anti-hypo-pHBc antibodies (extracellular). *F*, comparison of UBE2O- and Alix-mediated secretion of HBc or Ub-fused HBc variants. HepG2-NTCP cells were co-transfected with Flag-Myc-UBE2O or Flag-Alix expression plasmids together with various HBc and Ub-HBc variants, as indicated. The expression levels of UBE2O, Alix, HBc, and β-actin (loading control) were analyzed in the input samples (intracellular). The levels of secreted HBc and Ub-HBc were analyzed in the cell culture supernatants of transfected cells by Western blotting with anti-HBc, anti-hyper-pHBc and anti-hypo-pHBc antibodies (extracellular). Graph – Quantitative analysis of secreted HBc and Ub-HBc determined by ELISA in the cell supernatants. The data are presented as means ± SDs of one experiment performed in biological replicates (n = 3) shown by orange points. Statistical significance of differences between the control and UBE2O- or Alix-transfected groups was evaluated by a two-tailed *t* test (*p* ≥ 0.05 – not significant (ns); ∗∗*p* < 0.01; ∗∗∗*p* < 0.001).

Consistent with the proposed mechanism of UBE2O action, whereby client monoubiquitination triggers extensive multi-monoubiquitination mediated by UBE2O (38), the attachment of a single ubiquitin to various HBc variants (Ub-HBc-wt, Ub-HBc-Y132A, and Ub-HBc-S164A) resulted in their robust multi-monoubiquitination (Fig. 8*A*). Notably, the highest levels of multi-monoubiquitination were observed for the hypophosphorylated Ub-capsid (Ub-HBc-S164A) and monomeric Ub-HBc (Ub-HBc-Y132A) variants. Multi-monoubiquitination was further enhanced by the overexpression of UBE2O-wt (Fig. 8*A*; UBE2O upregulation). Conversely, the knockdown of endogenous UBE2O using UBE2O-specific siRNA significantly decreased the levels of monoubiquitinated and multi-monoubiquitinated HBc and Ub-HBc variants (Fig. 8*B*; UBE2O downregulation).

To validate that UBE2O catalyzes the multi-monoubiquitination of a pre-ubiquitinated core protein (Ub-HBc), we performed an *in vitro* ubiquitination assay designed to discriminate between polyubiquitination and multi-monoubiquitination (Fig. 8*C*). Similar to the experiment shown in Figure 5*C*, *in vitro* translated Ub-HBc was diluted 25-fold and incubated with an excess of either wild-type ubiquitin (Ub-wt, 5 μM) or chain-terminating methylated ubiquitin (Methyl-Ub, 5 μM). In reactions lacking recombinant UBE2O, incubation with Ub-wt yielded a high-molecular-weight smear (>100 kDa) (Fig. 8*C*, lane 4), consistent with polyubiquitination mediated by residual enzymatic activity in the lysate (39, 40). However, upon the addition of UBE2O, this smear was converted into distinct lower-molecular-weight bands ranging from 40 to 100 kDa (Fig. 8*C*, lane 5). Importantly, a similar ladder-like pattern was also observed when the reaction was performed with Methyl-Ub, which cannot support polyubiquitin chain assembly (Fig. 8*C*, lane 8). This finding allows us to exclude the possibility of polyubiquitination capped by methylated ubiquitin, as such a chain-capping mechanism would have resulted in a downward shift toward lower molecular-weight species due to premature termination of chain elongation. Instead, the identical laddering pattern observed with both wild-type and methylated ubiquitin provides evidence that UBE2O catalyzes multi-monoubiquitination, adding single ubiquitin moieties to multiple distinct sites on the HBc protein. Controls (Fig. 8*C*, lanes 1 and 2) support this interpretation, as the small residual ladder in reactions without added ubiquitin is consistent with the low levels of endogenous Ub in 1:25 RRL, which would not permit robust chain formation.

To confirm these findings in a cellular context, we co-expressed Ub-HBc and UBE2O in HepG2-NTCP cells along with either HA-tagged wild-type ubiquitin (HA-Ub-wt) or a lysine-less, chain-deficient mutant (HA-Ub-K0). Following immunoprecipitation of HA-tagged ubiquitinated proteins, we observed that co-expression of UBE2O with Ub-wt resulted in a smear of mono- and polyubiquitinated HBc (Fig. S7, lane 4). However, when chain elongation was prevented by using Ub-K0 (Fig. S7, lane 6), the polyubiquitination smear was eliminated and a distinct pattern of multi-monoubiquitinated HBc species (ranging from 40-100 kDa) became apparent, at levels comparable to the multi-monoubiquitination seen with wild-type ubiquitin (Fig. S7, compare lanes 4 and 6). Collectively, *in vitro* and cellular data provide strong evidence for a model in which the pre-conjugation of a single ubiquitin moiety to HBc facilitates its recognition by UBE2O, which then preferentially catalyzes multi-monoubiquitination rather than the extension of a polyubiquitin chain.

UBE2O-mediated enhancement of mono- and multi-monoubiquitination of HBc and Ub-HBc variants correlated with their increased secretion (Fig. 8*D*). The only exception to this pattern was the secretion observed for the hypophosphorylated HBc-Y132A variant, whose release was reduced by UBE2O overexpression (Fig. 8*D*, lane 7, WB: hypo-pHBc), a phenomenon already described in the preceding section. In contrast, UBE2O knockdown led to a significant overall reduction in the secretion of both HBc and Ub-HBc variants (Fig. 8*E*).

### Distinct roles of UBE2O and Alix in capsid trafficking and secretion: monoubiquitination-dependent *versus* -independent pathways

Alix was previously demonstrated to regulate the secretion of naked capsids independently of the ESCRT machinery and MVB pathway (33, 34). Therefore, to distinguish between UBE2O- and Alix-mediated secretory pathways, we examined the effects of these factors on the release of various capsid types, including wild-type (HBc-wt), phosphorylation-deficient (HBc-S164A), and their ubiquitin-fused counterparts (Ub-HBc-wt and Ub-HBc-S164A). While UBE2O was able to enhance the secretion of both capsids and their ubiquitin-fused variants, Alix specifically promoted the secretion of capsids but failed to stimulate the release of ubiquitin-fused capsids (Fig. 8*F*). These findings confirmed previous observations that naked capsid and virion secretion are regulated by two distinct pathways. One pathway involves UBE2O facilitating capsid maturation and envelopment through a mono- and multi-monoubiquitination-dependent mechanism, which is critical for virion assembly and release. The other pathway is regulated by Alix, which mediates naked capsid secretion *via* a mechanism that is independent of capsid monoubiquitination.

### UBE2O enzymatic activity stimulates virion production and secretion

**Figure 9:**
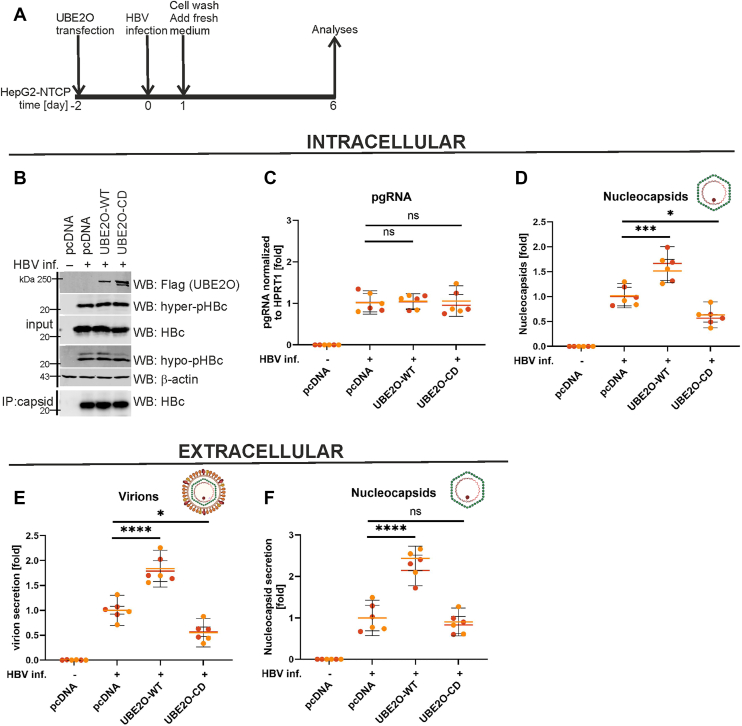
**Overexpression of UBE2O-WT enhances the secretion of both naked nucleocapsids and enveloped virions in HBV-infected HepG2-NTCP cells**. *A*, experimental design: HepG2-NTCP cells were transfected with either wild-type UBE2O (UBE2O-WT), catalytically-defective mutant (UBE2O-CD), or an empty vector (pcDNA). Two days post-transfection, the cells were infected with HBV. At 6 days post-infection, cells and culture supernatants were harvested to quantify intracellular pregenomic RNA (pgRNA), nucleocapsids, and the release of extracellular naked nucleocapsids and virions. *B*, protein extracts from HBV-infected HepG2-NTCP cells were subjected to Western blot analysis (input) and immunoprecipitation using anti-capsid antibodies (IP: capsid, WB: HBc). *C*, the levels of HBV pgRNA transcription were analyzed by RT-qPCR and normalized to the housekeeping gene, *HPRT1*. *D*, Analysis of intracellular DNA-containing nucleocapsids in HBV-infected HepG2-NTCP cells using immunoprecipitation with anti-capsids antibodies followed by qPCR. *E*, UBE2O-WT upregulation enhanced enveloped virion secretion, while the UBE2O-CD mutant reduced it. Viral particles were immunoprecipitated from the culture supernatants using anti-S antibodies. The associated DNA was then isolated and quantified by qPCR. *F*, UBE2O-WT upregulation enhanced the secretion of naked nucleocapsids. Nucleocapsids were immunoprecipitated from the culture supernatants using anti-capsid antibodies. The associated DNA was then isolated and quantified by qPCR. In (*C–F*), data are presented as the mean ± SD from two independent experiments. Color-coded data points distinguish individual replicates from each experiment, while colored horizontal lines indicate the mean values. Statistical significance of differences between UBE2O-WT, UBE2O-CD and control (pcDNA) groups was evaluated by 2-way ANOVA (*p* ≥ 0.05 – not significant (ns); ∗*p* < 0.05; ∗∗∗*p* < 0.001; ∗∗∗∗*p* < 0.0001).

To further investigate the role of UBE2O upregulation in HBV nucleocapsid assembly and the secretion of virions and naked nucleocapsids, we conducted analyses in HBV-infected HepG2-NTCP cells transfected with UBE2O expression plasmids (experimental layout shown in Fig. 9*A*). Overexpression of wild-type UBE2O (UBE2O-wt) had little impact on intracellular HBc and capsid levels (Fig. 9*B*) or pregenomic RNA transcription (Fig. 9*C*); however, it significantly increased the accumulation of DNA-containing capsids (Fig. 9*D*) and enhanced the secretion of both enveloped virions and naked nucleocapsids (Fig. 9, *E* and *F*). In contrast, overexpression of the catalytically defective UBE2O mutant (UBE2O-CD) resulted in a reduction of intracellular DNA-containing nucleocapsids (Fig. 9*D*), which was accompanied by decreased secretion of enveloped virions (Fig. 9*E*). These findings highlight the essential role of UBE2O's enzymatic activity in regulating HBV virion maturation and secretion.

## Discussion

The secretion of HBV is a highly regulated pathway that coordinates nucleocapsid assembly, maturation, envelopment, and egress. In addition to infectious virions, HBV produces and secretes large quantities of empty virions and naked nucleocapsids. In this study, we investigated the role of the E2/E3 hybrid Ub enzyme UBE2O in the HBV life cycle. Our findings demonstrate that UBE2O binds to and monoubiquitinates hypophosphorylated HBc and capsids (Figs. 1, 4*A*, 5, and 6). Notably, silencing of UBE2O expression in HBV-infected HepG2-NTCP cells or PHH significantly reduced intracellular nucleocapsid levels and attenuated virion secretion (Figs. 3 and S4). Conversely, overexpression of wild-type UBE2O (UBE2O-wt) increased the levels of intracellular nucleocapsids (Fig. 9*D*) and enhanced the secretion of both naked nucleocapsids and infectious virions (Fig. 9, *E* and *F*). Moreover, overexpression of catalytically defective UBE2O (UBE2O-CD) led to a decrease in intracellular nucleocapsids and inhibited the secretion of enveloped viral particles (Fig. 9, *D* and *E*). The transcription rate of pgRNA remained unchanged irrespective of the overexpression of either UBE2O-wt or UBE2O-CD (Fig. 9*C*).

While UBE2O knockdown led to a reduction of several markers of HBV replication, such as intracellular HBV DNA levels (Figs. 2*C* and S2*B*), pgRNA transcription (Figs. 2*E* and S2*D*), or the secretion of HBe antigen (Figs. 2*F* and S2*E*), cccDNA levels (Figs. 2*D* and S2*C*) remained unchanged. This observation aligns with the proposed role of UBE2O in the later stages of the HBV life cycle, particularly in capsid maturation and virion secretion. Since cccDNA formation is an early event involving capsid disassembly and nuclear import of relaxed circular DNA (rcDNA) (41), it is expected that UBE2O depletion would not impact cccDNA establishment and maintenance. In summary, our study identifies UBE2O as a critical host factor that HBV utilizes to regulate the balance between capsid maturation and virion secretion. By acting as both an E2 ubiquitin-conjugating enzyme and an E3 ubiquitin ligase, UBE2O modifies the core protein in a way that promotes its incorporation into productive viral particles. This establishes a direct link between a host ubiquitination enzyme and late stages of the HBV life cycle.

The findings presented here demonstrated that UBE2O interacts with both HBc monomers/dimers and assembled capsids (Fig. 1, *C*–*E*). While immunoprecipitation confirmed the interaction under endogenous expression conditions in HBV-infected cells (Fig. 1*D*), we additionally employed proximity-based techniques, such as proximity ligation assay (PLA) (Fig. 1, *A* and *B*) and immunofluorescence co-localization (Fig. 4*A*), to visualize and support the spatial association of UBE2O and HBc *in situ*.

*In vitro* ubiquitination and co-expression studies with capsid assembly-competent and -defective HBc variants revealed that UBE2O preferentially monoubiquitinates hypophosphorylated HBc and capsids (Figs. 5 and 6) residing in the cytoplasm (Fig. 5*A*). HBc hypophosphorylation is recognized as a hallmark of mature nucleocapsids. Phosphorylation and dephosphorylation act as molecular switches regulating HBc function. While hyperphosphorylated HBc is usually linked with immature or empty capsids, hypophosphorylation is necessary for packaging pregenomic RNA and completing reverse transcription, thereby designating nucleocapsids as replication-competent (11, 12, 42). We showed that HBc variants with single or multiple S-to-A substitutions in the C-terminal domain exhibited enhanced UBE2O-mediated monoubiquitination, with mutations at S164 and S172 resulting in the highest levels (Fig. 6, *A* and *B*). According to the “phosphorylation priming” model, phosphorylation at major SP sites (S157, S164, and S172) is necessary for phosphorylation at minor SQ sites (S178 and S180) (43). Therefore, we propose that S-to-A mutations at S164 and S172 are sufficient to induce hypophosphorylation of the HBc C-terminal domain, leading to maximal UBE2O-mediated monoubiquitination. Moreover, we have shown that monoubiquitinated HBc can be effectively assembled into capsid particles (Figs. 6*D*, 8*D*, and S6*C*), highlighting its functional relevance in HBV capsid biology.

A recent study by Yip *et al.* (38) identified two distinct mechanisms modulating UBE2O’s client selection: (i) ubiquitin-binding and (ii) the involvement of a nucleosome assembly protein 1-like one cofactor, NAP1L1. Their findings demonstrated that the addition of a single ubiquitin moiety to a client protein enhances both its interaction with UBE2O and the enzyme’s multi-monoubiquitination activity. Consistent with these observations, HBc/capsids failed to interact with a catalytically inactive mutant UBE2O-CD (Fig. 7*C*). However, fusing a single ubiquitin moiety to HBc (Ub-HBc) restored binding to UBE2O-CD (Fig. 7*C*), suggesting that the ubiquitin-binding SH3-C domain of UBE2O facilitates its association with monoubiquitinated HBc or capsids. Furthermore, the expression of various Ub-fused HBc variants in HepG2-NTCP cells led to their extensive multi-monoubiquitination, which was further modulated by UBE2O expression levels. Overexpression of UBE2O significantly enhanced multi-monoubiquitination, while its knockdown markedly reduced it (Fig. 8, *A* and *B*).

The multi-monoubiquitination activity of UBE2O was further demonstrated in an *in vitro* ubiquitination assay using ubiquitin-fused HBc (Ub-HBc) as a substrate and methylated ubiquitin as a reagent that terminates chain elongation (Fig. 8*C*). UBE2O generated an identical ladder of modified HBc species with both wild-type and methylated ubiquitin, which would not be possible if it were extending a polyubiquitin chain. Future work using a fully reconstituted system with HBc expressed in *E. coli* will be essential to unambiguously define the mechanistic basis of this modification.

**Figure 10:**
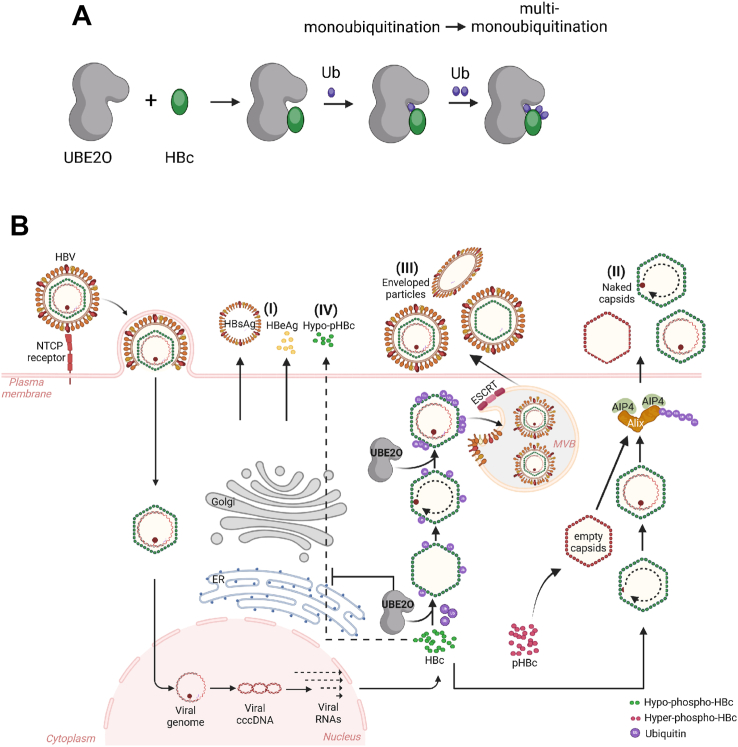
**Role of UBE2O in HBc/capsid ubiquitination, maturation and virion egress.***A*, Model for UBE2O-HBc binding (38). UBE2O-mediated monoubiquitination of HBc enhances the stability of the UBE2O-HBc complex, thereby promoting the subsequent multi-monoubiquitination of HBc. *B*, HBV infection results in the secretion of diverse viral progeny species, including: (I) HBeAg and spherical HBsAg; (II) naked capsids (NC, genome-positive and genome-negative); (III) infectious genome-positive HBV virions, empty genome-negative HBV virions, and non-infectious empty envelope particles (subviral particles, SVPs). (I) HBeAg and spherical HBsAg are secreted *via* the host ER-Golgi constitutive secretory pathway. (II) The egress of naked capsids, regardless of their nucleic acid content, is facilitated by Alix *via* an ESCRT-independent pathway. Interaction between Alix and the NEDD4 family E3 ubiquitin ligase AIP4 drives the release of HBV naked capsids. (III) Assembly and egress of infectious or empty virions rely on the ESCRT machinery, which facilitates capsid sorting into intraluminal vesicles (ILVs) within multivesicular bodies (MVBs). Host components of the MVB pathway support capsid envelopment and virion budding. Our findings demonstrate that UBE2O, an E2/E3 ubiquitin ligase, plays a critical role in these processes. UBE2O recognizes hypophosphorylated HBc and capsids, catalyzes their mono- and multi-monoubiquitination, and facilitates the efficient release of HBV virions. (IV) Hypophosphorylated unassembled HBc is also secreted, likely utilizing the ER-Golgi constitutive secretory pathway shared with HBeAg. Images were created in BioRender.com.

Based on the current data, we propose a model in which the initial binding of HBc to UBE2O triggers its monoubiquitination; this modification, in turn, enhances and stabilizes the UBE2O–HBc interaction, thereby promoting robust distributive multi-monoubiquitination (Fig. 10*A*). This proposed mechanism is consistent with the previously reported model of UBE2O binding to interferon-related developmental regulator 2 (IFRD2) (38). Although mass spectrometry identified NAP1L1 as a potential HBc-interacting partner (6), its role in facilitating the UBE2O-HBc interaction remains unclear and is currently under investigation.

Increased UBE2O levels were associated with enhanced capsid/HBc monoubiquitination and elevated secretion of these components (Figs. 7, *B* and *D* and 8*D*). In contrast, the loss of UBE2O resulted in reduced monoubiquitination and decreased secretion of HBc and capsids (Fig. 8*E*). The catalytically inactive UBE2O-CD mutant failed to promote HBc secretion and instead acted as a dominant-negative mutant (Fig. 7*D*). This led us to conclude that the enhancement of HBc secretion mediated by UBE2O is entirely dependent on its E2/E3 hybrid enzymatic activity. The functional significance of HBc monoubiquitination was further supported by the observation that while Ub-fused HBc and capsids were efficiently secreted from cells (Fig. S6*D*), they were unable to translocate to the nucleus (Fig. S6*C*).

The ubiquitination of HBc is considered to play a key role in the recognition of viral nucleocapsids by ESCRT components and their recruitment into MVBs. The ESCRT machinery normally functions in sorting cellular proteins into multivesicular bodies for secretion or degradation. HBV hijacks this pathway, using ubiquitin modifications on capsids as molecular signals to gain access to the host secretory system for virion release. HBc protein contains two potential ubiquitin acceptor lysine residues (K7 and K96) that are conserved across different HBV strains. While some studies have suggested that K96 may facilitate virion release (15, 16, 44, 45), likely through its conjugation to ubiquitin, the precise role of lysine ubiquitination in viral replication and virion egress remains controversial and requires further clarification. In addition to lysine residues, non-canonical sites such as serines or threonines are also potential targets for ubiquitination (46). The significance of ubiquitination at non-lysine residues may reflect the cell's ability to ubiquitinate proteins whose lysine residues are either inaccessible or absent (47). Our previous work provided direct mass spectrometry-based evidence that several serine and threonine residues in HBc can be ubiquitinated (5). In light of the findings presented in this study demonstrating that UBE2O mediates multi-monoubiquitination of HBc, it is reasonable to suggest that non-lysine residues may also serve as ubiquitination targets. A key future direction will be the precise mapping of the HBc residues targeted by UBE2O-mediated ubiquitination, which will require a detailed approach combining mass spectrometry analysis with extensive site-directed mutagenesis studies for validation.

Our study demonstrates that while the constitutive secretion of capsid-assembly-competent HBc is relatively low, capsid assembly-defective HBc variants secrete large quantities of hypophosphorylated HBc (Figs. 7*B* and 8, *D* and *E*). Given the sequence and structural similarities between HBeAg and hypophosphorylated HBc monomers, it is likely that both proteins utilize the same secretory pathway through the host ER-Golgi system. UBE2O overexpression appears to redirect hypophosphorylated HBc to the MVB/ESCRT pathway, potentially reducing its constitutive secretion and increasing its intracellular levels. Consistent with this hypothesis, HepG2-NTCP cells and PHHs with reduced UBE2O expression showed significantly lower intracellular levels of hypophosphorylated HBc (Figs. 3*A* and S4*A*) and nucleocapsids (Figs. 3*B* and S4*B*).

HBV capsid assembly is a highly concentration-dependent process, requiring a critical threshold of HBc monomers (48). Below this threshold, HBc monomers and dimers remain unassembled or form small, non-functional aggregates. A lack of sufficient HBc levels disrupts capsid assembly, reducing the efficiency of viral replication and pgRNA packaging (49), a phenomenon also observed here in UBE2O-deficient HepG2-NTCP cells (Figs. 2 and 3) or PHH (Figs. S2 and S4). Potential UBE2O's role in maintaining intracellular pools of hypophosphorylated HBc may be particularly advantageous during the early stages of HBV infection when the concentration of HBc is low. Once this critical concentration threshold is surpassed, assembly becomes highly cooperative, leading to efficient capsid formation (48, 50).

NEDD4 E3 ubiquitin ligase was recently identified as the factor that recognizes HBc *via* its PPAY motif and polyubiquitinates HBc at K96. Ubiquitinated HBc is then recognized by ESCRT component TSG101 and recruited to MVBs for virion envelopment (16). In our study, the overexpression of UBE2O led to an increased association between capsids and components of the ESCRT machinery, HGS, and SNF8/EAP30 (Fig. 4*G*). It is therefore possible that UBE2O-mediated mono- or multi-monoubiquitination of HBc may serve as a priming step for subsequent K63-linked polyubiquitination by NEDD4, which in turn promotes capsid recruitment to MVBs and facilitates virion secretion. This suggests a potential cooperative mechanism between UBE2O and NEDD4 in regulating HBc/capsid ubiquitination and trafficking. Further studies will be necessary to determine the precise hierarchy and interplay among these ubiquitination events.

Moreover, our data indicate that the knockdown of UBE2O impaired virion release (Figs. 3*C* and S4*C*) but did not affect the secretion of naked nucleocapsids (Figs. 3*D* and S4*D*). Notably, the overexpression of wt-UBE2O in HBV-infected cells led to an increase in intracellular nucleocapsid levels and enhanced the secretion of both enveloped virions and naked nucleocapsids, while having no direct effect on HBV RNA transcription (Fig. 9). This suggested that the elevated levels of UBE2O may promote the production of a surplus of functional nucleocapsids, which are subsequently directed to MVBs for efficient sorting into enveloped and non-enveloped secretory pathways, thereby enhancing the release of both particle types. It appears that the secretion of enveloped virions has a stricter dependency on optimal levels of UBE2O and other host factors, as it requires additional steps such as the interaction of nucleocapsids with HBV envelope proteins (HBsAg) and their envelopment by host-derived membranes. On the other hand, the release of naked nucleocapsids may bypass these processes, rendering it less dependent on other host factors. The observation that catalytically-defective UBE2O-CD exerts a dominant-negative effect on virion egress (Fig. 9*E*) but not on naked nucleocapsids release (Fig. 9*F*) suggests that virion secretion is more reliant on the Ub-ligase activity of UBE2O, particularly on capsid multi-monoubiquitination. This further indicates a critical role for UBE2O's enzymatic function in facilitating the processes specific to the envelopment and release of mature viral particles.

UBE2O was previously described as a pleiotropic protein with roles in several cellular processes. Although UBE2O was recently shown to facilitate endosome-to-Golgi retrograde transport by assisting the MAGE-L2:TRIM27 E3 Ub ligase complex in catalyzing K63-linked ubiquitination of the WASH protein (21), no direct evidence has established a connection between UBE2O and endosomal or other cellular compartments. Here, we demonstrate for the first time that UBE2O co-localizes with CD63-positive endosomes, approximately 0.5 μm in diameter, which contain ubiquitinated proteins characteristic of MVB-mediated secretion (Fig. 4, *C*–*F* and Video S1). Since ubiquitination is considered a key regulator of endosomal signaling, UBE2O's E2/E3 enzymatic activity may play a primary role in endosome trafficking, maturation, and sorting of cellular cargo proteins. UBE2O signals were also detected outside CD63-positive endosomes (Fig. 4, *C*–*F* and Video S1), suggesting that UBE2O shuttles between various cellular compartments and carries out multiple tasks depending on its location. The specific function of UBE2O in endosome sorting and exosome biogenesis requires further investigation.

Based on our findings and previously published data, we propose that HBV exploits UBE2O for nucleocapsid maturation and virion egress (Fig. 10*B*). UBE2O may fulfill a dual role in this process: (i) by regulating the host endosomal pathway, it facilitates virion maturation and release *via* MVBs; and (ii) through mono- and multi-monoubiquitination of hypophosphorylated HBc, UBE2O may promote capsid association with the ESCRT machinery or enhance its interaction with HBV envelope (HBs) proteins and host factors essential for envelopment. Conversely, the release of naked capsids is not dependent on capsid ubiquitination but is regulated by the collaborative action between Alix and NEDD4 family ubiquitin ligase AIP4 (34). In agreement with these findings, our study showed that Alix overexpression stimulated capsid secretion but not ubiquitin-fused capsid release (Fig. 8*F*). Thus, our data further demonstrate the diverse roles of the host ubiquitin machinery in regulating different HBV egress routes.

In conclusion, UBE2O is an important regulator of the host endosomal secretory pathway that HBV hijacks to support its replication and virion egress, demonstrating its critical role in the viral life cycle.

## Experimental procedures

### Cell lines

HepG2-NTCP (human liver cancer cell line HepG2, stably transfected with the human HBV entry receptor sodium taurocholate co-transporting polypeptide, NTCP) was obtained from Dr Stephan Urban (Heidelberg University Hospital). HepAD38, a HepG2-derived cell line that inducibly expresses the HBV genome under the control of a tet operator/CMV promoter, was kindly provided by Gilead Sciences. The cells were cultured with 0.3 μg/ml tetracycline during maintenance and passage; during the induction of HBV replication, they were cultured in the absence of tetracycline. Primary human hepatocytes (PHH) were purchased from BioIVT.

### Plasmids

The full-length wt-HBc (1–185 aa, HBV genotype A, subtype adw2) expression plasmid tagged with HA-tag was described previously (5). The His-Flag-HBc and HBc-Flag were generated by in-frame insertion of a His-Flag tag at the N-terminus or a Flag tag at the C-terminus of wt-HBc. The expression plasmid for wt-UBE2O tagged with Flag and Myc (wtUBE2O-F-M) was purchased from OriGene Technologies. The Flag-tagged Alix expression plasmid (Alix-F, Addgene plasmid #89859) was a gift from Wesley Sundquist (51). The capsid assembly-competent (HBc-Y132F), capsid assembly-defective (HBc-Y132A and HBc-W102A) variants, S-to-A mutants of HBc and the catalytically-defective C1040S mutant of UBE2O (UBE2O-CD-F-M) were generated by PCR, using QuickChange XL Site-Directed Mutagenesis (Agilent Technologies). The HBc deletion mutants (in Fig. S1*D*), HBc N-terminal (aa 39–185 and 92–185), C-terminal truncations of HBc (aa 1–134, 1–140 and 1–150), or internal deletions (del134–149 and del93–149), were all tagged with an HA tag at the C-terminus and were generated by PCR, using the QuickChange XL Site-Directed Mutagenesis Kit (Agilent Technologies). To generate the ubiquitin-fusion HBc construct (Ub-HBc-wt), ubiquitin (G76V) was inserted at the N-terminal part of the HBc open reading frame (ORF) by Gibson assembly cloning. Ub-HBc mutants, including Ub-HBc-Y132A and Ub-HBc-S164, were generated by PCR amplification of Ub-HBc-wt, using QuickChange XL Site-Directed Mutagenesis (Agilent Technologies). The fidelity of all constructs was verified by Sanger sequencing (Seqme).

The following HA-tagged ubiquitin expression plasmids were gifts from Ted Dawson (52): pRK5-HA-Ubiquitin-wt (Ub-wt, Addgene plasmid #17608), pRK5-HA-Ubiquitin-K33 (Ub-K33, Addgene plasmid #17607), pRK5-HA-Ubiquitin-K48 (Ub-K48, Addgene plasmid #17605), pRK5-HA-Ubiquitin-K63 (Ub-K63, Addgene plasmid #17606), pRK5-HA-Ubiquitin-K0 (Ub-K0, Addgene plasmid #17603), pRK5-HA-Ubiquitin-K29R (Ub-K29R, Addgene plasmid #17602) and pRK5-HA-Ubiquitin-K48R (Ub-K48R, Addgene plasmid #17604); from Sandra Weller (53): pRK5-HA-Ubiquitin-K6 (Ub-K6, Addgene plasmid #22900), pRK5-HA-Ubiquitin-K11 (Ub-K11, Addgene plasmid #22901), pRK5-HA-Ubiquitin-K27 (Ub-K27, Addgene plasmid #22902), pRK5-HA-Ubiquitin-K29 (Ub-K29, Addgene plasmid #22903), and from Josef Kittler (54): pRK5-HA-Ubiquitin-K6R (Ub-K6R, Addgene plasmid #121153), pRK5-HA-Ubiquitin-K11R (Ub-K11R, Addgene plasmid #121154), pRK5-HA-Ubiquitin-K27R (Ub-K27R, Addgene plasmid #121155). Plasmids pRK5-HA-Ubiquitin-K33R (Ub-K33R) and pRK5-HA-Ubiquitin-K63R (Ub-K63R) were described previously (6).

### Antibodies

All antibodies used in the study are listed in Table S1. The primary antibodies and reagents used for the Western blotting and immunoprecipitations included antibodies against UBE2O (Novus Biologicals), ubiquitin-HRP (BioLegend), HBV surface antigen (S14, Abcam), β-actin (Abcam), Flag (Sigma-Aldrich), HA (Sigma-Aldrich), α-tubulin (Sigma-Aldrich, USA), lamin A/C (Santa Cruz Biotechnologies), and anti-HA magnetic beads (ThermoFisher Scientific, USA). Antibodies against total HBc (anti-HBc; GS-1051053), hyperphosphorylated HBc (anti-hyper-pHBc; GS-1051052) and hypophosphorylated-HBc (anti-hypo-pHBc; GS-1051054) (all rabbit monoclonal) were obtained from Dr Rudolf K. Beran (Gilead Sciences, Inc). Mouse monoclonal antibodies specific against HBV capsids (Hyb-3120) were purchased from the Institute of Immunology Co., LTD. Secondary antibodies included goat polyclonal anti-mouse conjugated with horseradish peroxidase (HRP) (Sigma-Aldrich) and goat polyclonal anti-rabbit–HRP (Sigma-Aldrich). For visualization, we used a SuperSignal West Femto Maximum Sensitivity Substrate (ThermoFisher Scientific) and an LAS-4000 imager. As secondary antibodies for the LI-COR system, we used goat anti-rabbit IgG (H+L) IRDye 800CW (LI-COR Biosciences) and goat anti-mouse IgG (H+L) IRDye 680RD (LI-COR Biosciences). The Western blots were visualized using an LI-COR Odyssey CLx system and Image Studio Lite Software. The antibodies used for the immunofluorescence assay (IFA) and proximity ligation assay (PLA) were the following: mouse monoclonal antibodies against HBV capsids (Hyb-3120) (Institute of Immunology Co, LTD), rabbit monoclonal antibody against hypophosphorylated-HBc (hypo-pHBc) (a gift from R. Beran), rabbit polyclonal antibody against UBE2O (ThermoFisher Scientific), rabbit monoclonal against CD63 (EPR22458–280) and mouse monoclonal antibody against CD63 (MX-49.129.5) (both from Abcam), rabbit monoclonal antibody against TSG101 (Abcam), and mouse antibody against ubiquitinated proteins (clone FK2, Millipore).

### siRNAs

All siRNAs were purchased from ThermoFisher Scientific. The siRNAs specific to UBE2O were s34219 (si_UBE2O-A), s34220 (si_UBE2O-B) and s34221 (si_UBE2O-C). Two non-targeting Silencer Select Negative Control siRNAs, No. One and No. 4, were used as negative controls designated si_ctrl-A and si_ctrl-B, respectively. The siRNAs were transfected using Lipofectamine RNAiMAX (ThermoFisher Scientific), according to the manufacturer’s recommendations. The specificity and inhibitory potential of UBE2O-siRNAs was estimated by RT-qPCR using the primer pairs UBE2O-F and UBE2O-R (Table S2). RNA was isolated from transfected HepG2-NTCP cells or primary human hepatocytes (PHH) using the RNeasy Plus kit (Qiagen) and treated with dsDNase (ThermoFisher Scientific) according to the manufacturer’s recommendations. The RNAs were then analyzed by RT-qPCR using the Luna Universal One-Step RT-qPCR Kit (NEB) and real-time PCR cycler CFX Opus 96. The levels of *UBE2O* mRNA were normalized against the housekeeping gene hypoxanthine phosphoribosyltransferase 1 (*HPRT1*). The primers used for the amplification of *HPRT1* cDNA, HPRT1-F and HPRT1-R, are listed in Table S2.

### Validation of anti-capsid (Hyb-3120) antibody by a particle gel assay

HepG2-NTCP cells were transfected with various capsid assembly-competent and defective variants. Two days post-transfection the cell lysates were isolated, and the intracellular capsids were analyzed with particle gel assay as described previously (55).

### Preparation of the hepatitis B virus

The HepAD38 cell line was used for HBV production and purification. Infectious particles (Dane particles) were purified by 6% PEG-precipitation and centrifugation from collected cell-free supernatants.

### HBV infection of HepG2-NTCP cells and PHH

Two days before infection, the HepG2-NTCP cells were incubated in a medium supplemented with 2.5% DMSO. The cells were infected with HepAD38-derived HBV [the multiplicity of infection (MOI) was 1000 viral genome equivalents (VGE) per cell] in the presence of 4% PEG8000 overnight. 16 hours later, the cells were washed 6 times with PBS followed by the addition of fresh DMEM medium supplemented with 2.5% DMSO.

PHHs were infected with HepAD38-derived HBV (MOI was 500 VGE per cell) overnight in the presence of 4% PEG8000. Then, PHHs were washed 6 times with the Williams E Medium (ThermoFisher Scientific) and maintained in the Williams E medium supplemented with the Primary Hepatocyte Maintenance Supplements kit (ThermoFisher Scientific) and 1.5% DMSO. Six days post-infection (PI), the cells and medium were harvested for further analyses.

### HBV pgRNA quantification

Six days post-infection, the cells were harvested for RNA isolation using the RNeasy Plus Mini Kit (Qiagen). Contaminating genomic DNA was removed by treatment with dsDNase (ThermoFisher Scientific) according to the manufacturer’s recommendations. RNA integrity was evaluated by agarose gel electrophoresis. RNA was reverse-transcribed and amplified using the Luna Universal One-Step RT-qPCR Kit (NEB) with the primers pgRNA-F and pgRNA-R (Table S2) on a CFX Opus 96 real-time PCR cycler (Bio-Rad). The levels of pgRNA were normalized against the housekeeping gene hypoxanthine phosphoribosyltransferase 1 (*HPRT1*). The primers used for the amplification of *HPRT1* cDNA, HPRT1-F and HPRT1-R are listed in Table S2.

### Total HBV DNA and cccDNA quantification

Six days post-infection, total cellular DNA was isolated from HBV-infected HepG2-NTCP and PHHs with the NucleoSpin Tissue Kit (Macherey–Nagel, Germany). The total HBV DNA level was determined by quantitative PCR (qPCR) using the gb Elite PCR Master Mix (Generi Biotech, Czech Republic) with primers and a probe specific for HBV DNA, HBV-F, HBV-R and PROB-HBV (Table S2) (5). The level of HBV DNA was normalized against albumin using primers Alb-F and Alb-R and probe PROB-Alb (Table S2). cccDNA quantification was performed as described previously (56, 57). Briefly, 1 μg of DNA was treated with 10 units of T5 exonuclease (NEB) for 2 h. Then, DNA was purified using the DNA Clean and Concentrator Kit (Zymo Research). qPCR was performed with the gb Elite PCR Master Mix (Generi Biotech) and cccDNA-specific primers and probe – ccc-F, ccc-R and PROB-ccc. The level of cccDNA was normalized against mitochondrial-encoded cytochrome-c oxidase subunit II (*MT-CO2*) in samples without T5 exonuclease digestion. cccDNA-specific and MT-CO2-specific primers and probes (ccc-F, ccc-R, MTCO2-F, MTCO2-R, PROB-ccc, PROB-MTCO2) are listed in Table S2. The effectiveness of T5 treatment and the specificity of cccDNA primers were validated by comparing T5-treated and untreated samples, and by analyzing cccDNA-specific primers against total HBV DNA primers (non-specific for cccDNA).

### HBeAg and HBc detection by ELISA

The titers of HBeAg or HBc secreted by HBV-infected or HBc-transfected cells were quantified by ELISA. HepG2-NTCP or PHH cell culture supernatants were collected and centrifuged at 120 *g* for 10 min to remove cellular debris, transferred to clean tubes and stored at −80 °C until the antigen measurement. The titers of HBeAg or HBc were measured using a commercial ELISA kit (Bioneovan) according to the manufacturer’s instructions.

### Quantification of DNA associated with intracellular nucleocapsids

On day six post-infection, HepG2-NTCP and PHH cells were lysed in a lysis buffer containing 20 mM Tris (pH 7.6), 0.1 mM EDTA, 2 mM MgCl_2_, 0.5% Igepal, supplemented with 1 mM PMSF, 0.2 mM protease inhibitor cocktail (Merck Millipore) and phosphatase inhibitor mix I (Serva). 50 micrograms of protein samples were treated with 30 U of micrococcal nuclease (Merck, Germany) and 10 U of DNase I RNase-free (ThermoFisher Scientific, USA) at 37 °C for 90 min (to remove free RNA and DNA), followed by the addition of 10 mM EDTA. The lysates were then immunoprecipitated with anti-capsid antibodies (Hyb-3120) followed by incubation with Pierce Protein A/G Magnetic Beads (ThermoFisher Scientific, USA) at 4 °C overnight. The immunoprecipitated complexes were extensively washed with the lysis buffer, and the nucleocapsid-associated DNA was purified using the Purelink viral RNA/DNA mini kit (ThermoFisher Scientific) according to the manufacturer’s recommendations. To quantify DNA-containing nucleocapsids, the purified DNA samples were subjected to quantification by qPCR using the LightCycler 480 SYBR Green I Master kit (Roche Diagnostics) and two sets of primers: HBV-F and HBV-R (located in the HBx ORF), and pgRNA-F and pgRNA-R (located in the pgRNA promoter region) (Table S2). The results obtained with the two sets of primers were compared and turned out to be comparable. The presented graphical analyses are therefore based on the results obtained with the pgRNA primer set.

### Quantification of secreted naked nucleocapsids and enveloped virions

The analysis of HBV particle secretion was performed according to a previously described protocol (58), with minor modifications. HepG2-NTCP or PHH cells were transfected with siRNAs or expression plasmids. Two days after transfection, the cells were infected with HBV. The following day, the cells were extensively washed six times with large volumes of 1xPBS to effectively remove the HBV inoculum. On day six post-infection, 200 μl of cell culture supernatants were collected and clarified by centrifugation at 500*g* for 10 min and then treated with 30 U of micrococcal nuclease (Merck) and 10 U of DNase I RNase-free (ThermoFisher Scientific) at 37 °C for 90 min (to remove free RNA and DNA) followed by the addition of 10 mM EDTA. Secreted naked nucleocapsids and enveloped virions were precipitated with anti-capsid (Hyb-3120, Japan) or anti-S (S-14, Abcam, UK) antibodies, respectively, followed by incubation with Pierce Protein A/G Magnetic Beads (ThermoFisher Scientific) at 4 °C overnight. The immunoprecipitated complexes were extensively washed with the lysis buffer supplemented with 0.5% Igepal, and viral particle-associated DNA was purified using the Purelink viral RNA/DNA mini kit (ThermoFisher Scientific) according to the manufacturer’s recommendations. The purified DNA samples were subjected to quantification by qPCR using LightCycler 480 SYBR Green I Master kit (Roche Diagnostics) and two sets of primers: HBV-F, HBV-R and pgRNA-F, pgRNA-R (Table S2). As with the quantification of intracellular nucleocapsids, the results obtained with both sets of primers were comparable, so the graphical analyses presented in this study are based on the data obtained with the pgRNA primer pair.

### Transfection

All transfections with expression plasmids or siRNAs were performed using Lipofectamine 3000 or Lipofectamine RNAiMAX transfection reagents, respectively (both from ThermoFisher Scientific). HepG2-NTCP cells were transfected on 24-well plates, 6-well plates, or T75 flask formats. In all transfection experiments, the total amount of transfected DNA or siRNA was kept constant across all samples by supplementing with empty vector DNA (pcDNA) or control siRNA (si_ctrl-A), respectively.

### Preparation of protein samples

For nuclear and cytoplasmic extract preparation, cells were resuspended in five packed cell volumes of buffer A containing 20 mM Tris (pH 7.6), 0.1 mM EDTA, 2 mM MgCl_2_ supplemented with 1 mM phenylmethylsulfonyl fluoride (PMSF), 0.2 mM protease inhibitor cocktail (Sigma-Aldrich) and phosphatase inhibitor mix I (Serva). The cells were incubated for 10 min on ice. Thereafter, CHAPS (Merck Millipore, USA) was added at a final concentration of 0.6% (v/v), and the lysates were homogenized by being passed through a 20-gauge needle three times. Nuclei were pelleted by centrifugation at 600*g* for 5 min at 4 °C, and the supernatant containing cytoplasmic proteins was collected and stored at −80 °C. The remaining nuclei were washed three times in buffer A containing 0.6% CHAPS. The nucleic pellets were lysed in buffer B containing 20 mM HEPES (pH 7.9), 0.4 M NaCl, 2.5% glycerol, 1 mM EDTA, 1 mM PMSF, 0.5 mM DTT, supplemented with 0.2 mM protease inhibitor cocktail (Merck Millipore) and phosphatase inhibitor mix I (Serva, Germany) by repeated freezing and thawing. Supernatants containing soluble nucleic proteins were collected by centrifugation at 20,000 *g* for 20 min and stored at −80 °C.

For HBc, UBE2O and capsid co-immunoprecipitation experiments, the cells were lysed in a co-immunoprecipitation buffer (20 mM HEPES, pH 7.6, 100 mM NaCl, 1 mM EDTA, 10% glycerol, 0.5% Igepal) supplemented with 1 mM PMSF, and 0.2 mM protease inhibitor cocktail [all Sigma-Aldrich]). Protein extracts (50–400 μg) were incubated with anti-HA magnetic beads (ThermoFisher Scientific) or anti-capsid (Hyb-3120) and anti-Flag antibodies, followed by Protein A/G magnetic beads (ThermoFisher Scientific, USA) incubation at 4 °C overnight. Immune complexes were extensively washed with co-immunoprecipitation buffer supplemented with 1% Igepal and analyzed by Western blotting with anti-Flag or anti-HBc, -hyper-pHBc, and -hypo-pHBc antibodies.

For the detection of HBc ubiquitination, the cells were lysed in 2% SDS, 150 mM NaCl, 10 mM Tris-HCl (pH 8.0) with 2 mM sodium orthovanadate, 50 mM sodium fluoride, 10 mM N-ethylmaleimide (NEM) and protease inhibitors, followed by incubation at 100 °C for 10 min and sonication. The lysates were then diluted with nine volumes of dilution buffer (10 mM Tris-HCl, pH 8.0, 150 mM NaCl, 2 mM EDTA, 1% Triton X-100) and subjected to immunoprecipitation with anti-HA magnetic beads (ThermoFisher Scientific) followed by incubation with Pierce Protein A/G Magnetic Beads (ThermoFisher Scientific). After overnight incubation at 4 °C, the precipitates were washed with the washing buffer (10 mM Tris-HCl, pH 8.0, 1 M NaCl, 1 mM EDTA, 1% Igepal CA-630) and analyzed by Western blotting with anti-HBc, -hyper-pHBc, or -hypo-pHBc antibodies.

Due to the low levels of hypophosphorylated HBc in HBc-transfected or HBV-infected cells, we employed four times the amount of protein lysates for the Western blot analysis of hypophosphorylated HBc compared to the analysis of total or hyperphosphorylated HBc in input samples.

### *In vitro* ubiquitination

Wild-type HBc (HBc-wt) and its N-terminal ubiquitin-fused variant (Ub–HBc-wt) were synthesized *in vitro* using the TNT Coupled Reticulocyte Lysate System (Promega), supplemented with unlabeled methionine, following the manufacturer's protocol. Translation reactions were carried out for 2 h at 37°C. For *in vitro* ubiquitination, 1 μl of each translation product was incubated in a total reaction volume of 25 μl with components from the Ubiquitination assay kit (Abcam), including 75 nM E1 enzyme, 400 nM UBE2O (Abcam) and either 5 μM recombinant ubiquitin or methylated ubiquitin (Enzo Life Sciences). Reactions were incubated at 37°C for 3 h. Following incubation, samples were denatured in protein sample buffer, resolved by SDS-PAGE, and analyzed by Western blotting using anti-HBc antibodies.

### Immunofluorescence assay (IFA) and confocal microscopy

Cells were washed with PBS and fixed with 4% formaldehyde in PBS for 15 min. Cells were then permeabilized with 0.5% Triton X-100 in PBS for 5 min, washed 3× with PBS and blocked with 1% BSA for 1 h at room temperature. Specific primary antibodies were diluted in 1% BSA and were added for 1 h at room temperature. Donkey anti-rabbit conjugated to Alexa Fluor 488 and goat anti-mouse conjugated to Alexa Fluor 594 were used as secondary antibodies. Cell membranes and nuclei were stained with Abberior STAR ORANGE membrane probe (Abberior GmbH) and DAPI, respectively. Cells were then mounted on droplets of Anti-Fade Fluorescence Mounting Medium (Abcam). Images were captured using an LSM 880NLO confocal microscope (Carl Zeiss) and an IXplore SpinSR SoRa confocal microscope (Olympus).

### Proximity ligation assay (PLA)

PLA assay was performed using the Duolink Proximity ligation assay kit (Merck) according to the manufacturer's instructions with minor modifications. Briefly, cells were fixed on coverslips with 4% paraformaldehyde in PBS for 15 min and permeabilized with 0.5% Triton X-100 in PBS for 5 min. Cells were blocked with 3% BSA for 45 min and then incubated with primary antibodies diluted in 1% BSA overnight at 4 °C. Anti-rabbit plus (DUO92002) and anti-mouse minus (DUO92004) probes were applied, followed by a ligation reaction and rolling circle amplification. DAPI was used to stain cell nuclei, and the cells were mounted on droplets of Anti-Fade Fluorescence Mounting Medium (Abcam). Detection probes containing a fluorophore (ex. = 495 nm and em. = 527 nm) were visualized using fluorescence microscopy.

### Cytotoxicity assay

HepG2-NTCP cells were transfected with two control (si_ctrl-A and -B) and three UBE2O-specific (si_UBE2O-A, -B, and -C) siRNAs. Cell viability was determined on days 4, 6 and 8 post-transfection using a formazan-based proliferation assay (XTT assay) (59).

### Statistical analysis

Statistical analyses were performed in GraphPad Prism version 9.1.2 (GraphPad Software). Results in graphs are expressed as means ± SD. The Shapiro-Wilk test was used to assess whether data with multiple samples were normally distributed. The statistical significance of differences between samples was analyzed with a two-tailed *t* test or 2-way ANOVA followed by Tukey’s *post hoc* test (*p* ≥ 0.05 – not significant (ns); ∗*p* < 0.05; ∗∗*p* < 0.01; ∗∗∗*p* < 0.001; ∗∗∗∗*p* < 0.0001).

## Data availability

The data supporting the findings of this study are available upon request from the corresponding author.

## Supporting information

This article contains supporting information.

## Conflict of interest

The authors declare that they do not have any conflicts of interest with the content of this article.
