# Supplementary material for: UBE2O, a host ubiquitin-conjugating enzyme, is a key regulator of hepatitis B virus maturation and egress
Source: J Biol Chem. 2025 Sep 22;301(11):110750. doi: 10.1016/j.jbc.2025.110750 (PMC12554184; doi:10.1016/j.jbc.2025.110750)
Supplement: Supporting information [file mmc1.docx]

**Supporting information**

**Title**

**UBE2O, a host ubiquitin-conjugating enzyme, is a key regulator of Hepatitis B virus maturation and egress**

**Short title**

**UBE2O is required for HBV maturation and egress**

Barbora Lubyova^1^, Eva Tikalova^1^, Vaclav Janovec^1, 2^, Boris Ryabchenko^2^, Kristyna Krulova^1,3^, Vaclav Kropacek^1,3^, Sandra Huerfano^2^, Ivan Hirsch^1, 2^ and Jan Weber^1, *^

^1^ Institute of Organic Chemistry and Biochemistry of the Czech Academy of Sciences, Prague, Czech Republic,

^2^ Department of Genetics and Microbiology, Faculty of Science, Charles University, BIOCEV, Vestec, Czech Republic,

^3^ Department of Genetics and Microbiology, Faculty of Science, Charles University, Prague, Czech Republic

^*^ Corresponding author

**Tables**

**Table S1. List of antibodies**

**Table S2. List of primers and probes**

| **Primer** | **Sequence (5’ to 3’)** |
| --- | --- |
| UBE2O-F | CACATGCGATCCACCGACAG |
| UBE2O-R | CAGCCAGCAGTCATAGGCAA |
| pgRNA-F | CACCTCTGCCTAATCATC |
| pgRNA-R | GGAAAGAAGTCAGAAGGCAA |
| HBV-F | AGAGGACTCTTGGACTCTCAGC |
| HBV-R | CTCCCAGTCTTTAAACAAACAGTC |
| ccc-F | CCGTGTGCACTTCGCTTCA |
| ccc-R | GCACAGCTTGGAGGCTTGA |
| HPRT1-F | CCTGGCGTCGTGATTAGTGAT |
| HPRT1-R | GGGCTACAATGTGATGGCCT |
| Alb-F | GCTGTCATCTCTTGTGGGCTGT |
| Alb-R | AAACTCATGGGAGCTGCTGGTT |
| MTCO2-F | TGCCCGCCATCATCCTA |
| MTCO2-R | CGTCTGTTATGTAAAGGATGCGT |
| **Probe** |  |
| PROB-HBV | [FAM]-TCAACGACCGACCTT-[BHQ1] |
| PROB-ccc | [FAM]-CATGGAGACCACCGTGAACGCCC-[BHQ1] |
| PROB-Alb | [FAM]-GGAGAGATTTGTGTGGGCATGACAGG-[BHQ1] |
| PROB-MTCO2 | [FAM]-TCCTCATCGCCCTCCCATCCC-[BHQ1] |

**Figures and Figure Legends**

**Figure S1**

**
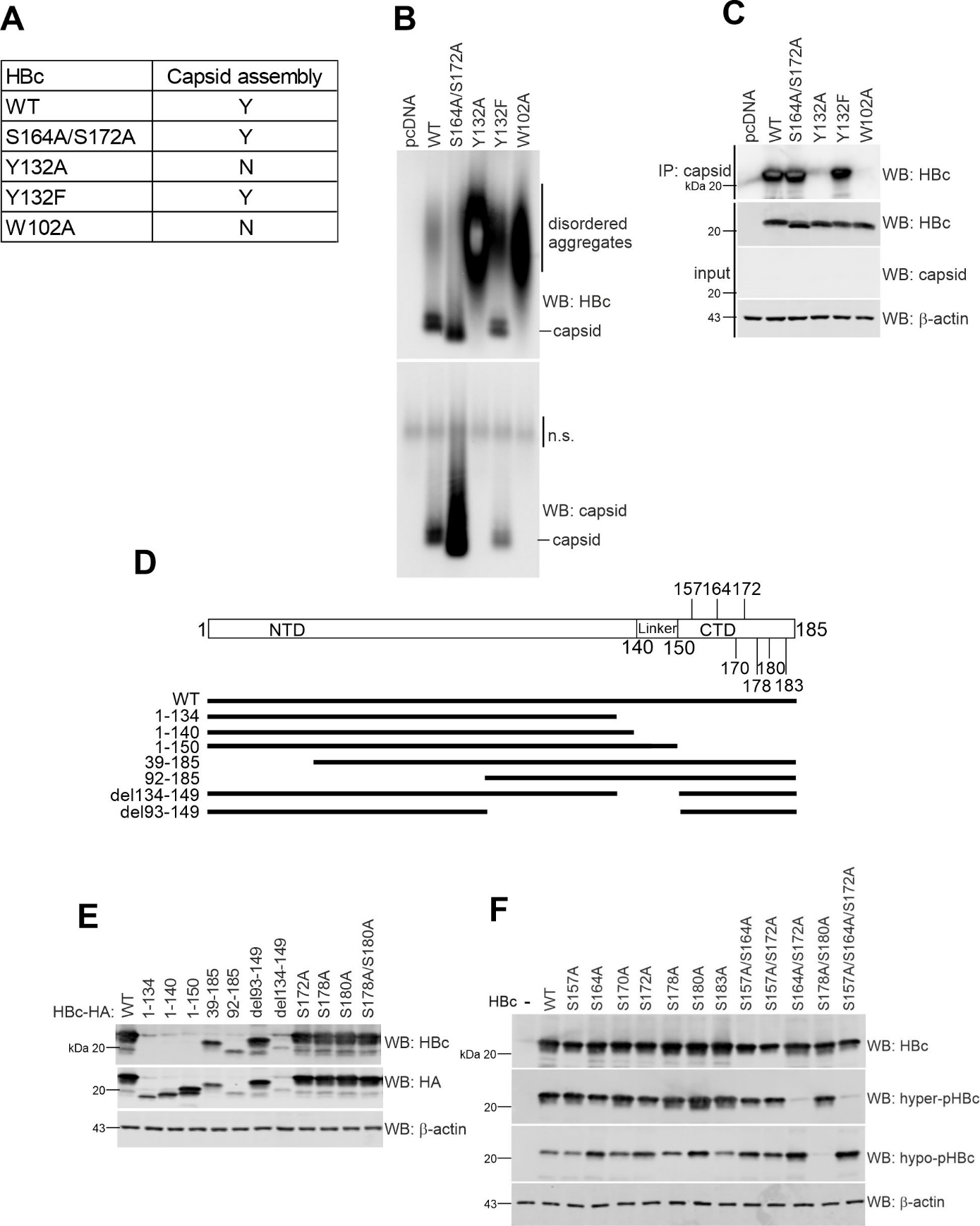
**

**Figure S1. Validation of anti-capsid (Hyb-3120) and anti-HBc antibodies.**

**(A-C)** Validation of anti-capsid (Hyb-3120) antibodies using particle gel assay. **(A)** Previously published capsid assembly properties of HBc wt and mutant variants. Y, capsid assembly-competent; N, capsid assembly-defective. **(B, C)** HepG2-NTCP cells were transfected with capsid assembly-competent (wt, S164A/S172A, and Y132F) and capsid assembly-defective (Y132A and W102A) HBc variants. **(B)** Protein lysates from transfected cells were analyzed by particle gel assay using anti-HBc and anti-capsid (Hyb-3120) antibodies. Note: The particle gel assay shown here is an agarose-based native gel system used to analyze viral capsid-containing structures in cell lysates. This method preserves the native conformation of HBV core particles, allowing detection by anti-capsid antibodies. **(C)** The same lysates were immunoprecipitated (IP) with anti-capsid antibodies and subsequently examined by Western blot (WB) using anti-HBc antibodies. Western blot analysis with anti-capsid antibodies (input) showed no signal, confirming their specificity for the native capsid conformation. HBc and β-actin expression levels are shown for comparison (input). **(D-F)** Epitope mapping of three anti-HBc antibodies used in the study. **(D)** Schematic representation of HBc and its deletion mutants. **(E)** Anti-HBc antibodies that recognize the HBc protein regardless of its phosphorylation status bind to the C-terminal domain (aa 150–185). HepG2-NTCP cells were transfected with either wt-HBc or various deletion and S-to-A HBc mutants. Forty-eight hours after transfection, the cells were harvested and protein lysates were analyzed by Western blotting with anti-HBc antibodies. **(F)** Anti-hyperphosphorylated HBc (hyper-pHBc) antibodies recognize the phosphorylated epitope S164–S172. Anti-hypophosphorylated HBc (hypo-pHBc) antibodies bind to the non-phosphorylated epitope of S178–S180. HepG2-NTCP cells were transfected with wt-HBc or various single and multiple S-to-A HBc mutants. Forty-eight hours after transfection, the cells were harvested and the protein lysates were analyzed by Western blotting with anti-HBc, anti-hyper-pHBc or anti-hypo-pHBc antibodies.

**Figure S2**

**
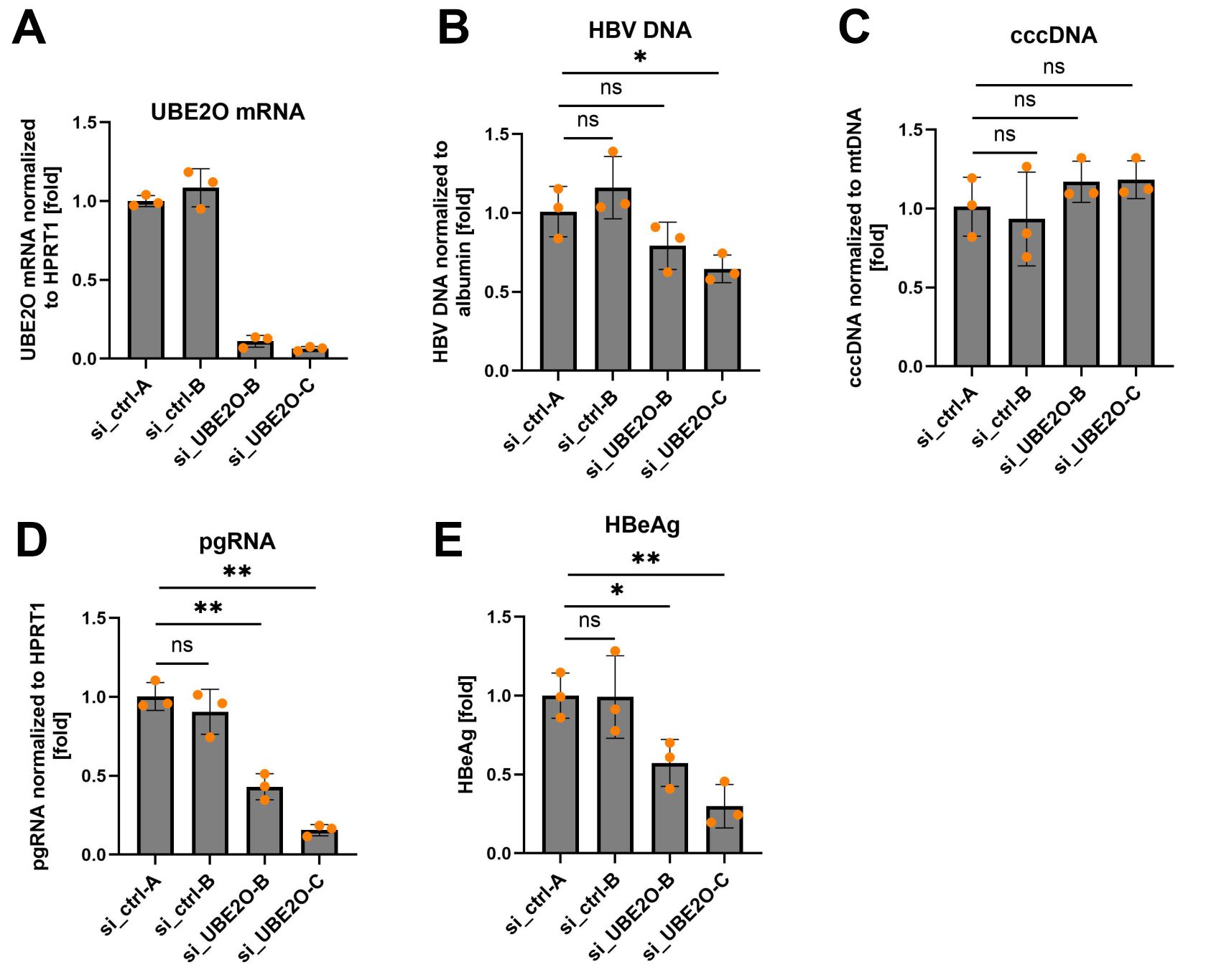
**

**Figure S2. Inhibition of UBE2O expression reduces HBV replication and secretion of HBe antigen in HBV-infected** **primary human hepatocytes (PHH).**

**(A-E)** PHH were transfected with two UBE2O-specific siRNAs (si_UBE2O-B, -C) or two control siRNAs (si_ctrl-A, -B) and then infected with HBV, following the experimental outline shown in Figure 2A. **(A)** Specificity and knockdown efficiency of UBE2O-specific siRNAs in PHH were analyzed by RT-qPCR. The levels of *UBE2O* mRNA were normalized against the housekeeping gene *HPRT1*.  **(B)** The levels of total HBV DNA in HBV-infected PHH were determined by qPCR and normalized to albumin. **(C)** The levels of cccDNA were determined by qPCR and normalized to mitochondrial DNA. **(D)** The levels of HBV pregenomic (pg) RNA transcription were analyzed by RT-qPCR and normalized to the housekeeping gene, *HPRT1*. **(E)** The levels of HBeAg in the cell culture supernatants of HBV-infected PHH were analyzed by enzyme-linked immunosorbent assay (ELISA). In **(A-E)**, the data are presented as means ± SDs of one experiment performed in biological replicates (n = 3) shown by orange points. Statistical significance of differences between UBE2O silencing and control (si_ctrl-A) groups was evaluated by a two-tailed t-test (p ≥ 0.05 – not significant (ns); * p < 0.05; ** p < 0.01).

**Figure S3**

**
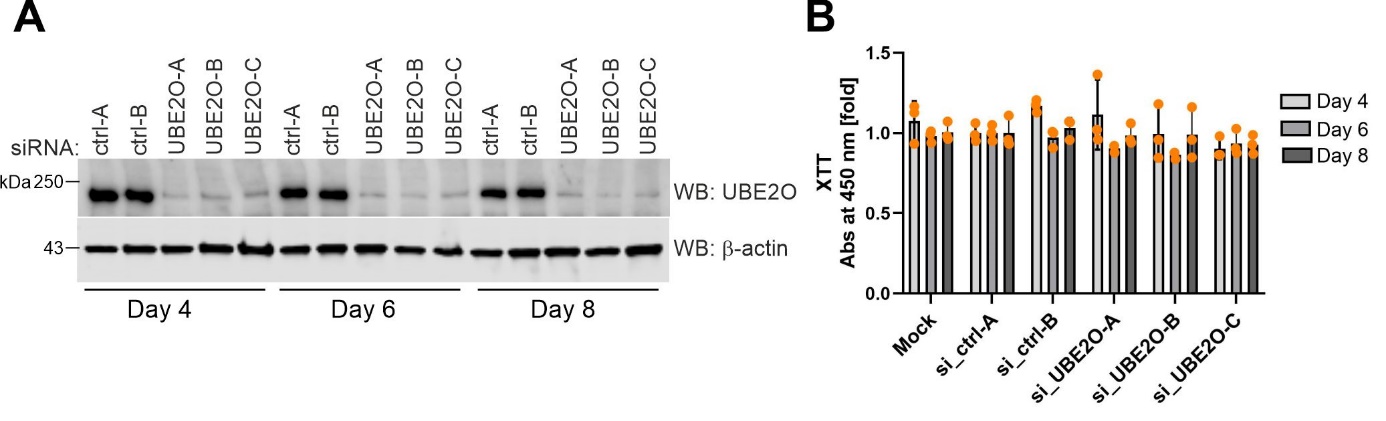
**

**Figure S3. Specificity, knockdown efficiency, and non-toxicity of control and UBE2O-specific siRNAs in HepG2-NTCP cells.**

**(A)** HepG2-NTCP cells were transfected with two control (si_ctrl-A and si_ctrl-B) or three UBE2O-specific (si_UBE2O-A, si_UBE2O-B and si_UBE2O-C) siRNAs and 4, 6 and 8 days post-transfection the protein lysates were prepared and analyzed by Western blotting with anti-UBE2O and anti-β-actin antibodies. **(B)** XTT assay to determine the level of potential cytotoxicity of siRNAs transfected into HepG2-NTCP cells at 4, 6, and 8 days after transfection. The data are presented as means ± SDs of one experiment performed in biological replicates (n = 3) shown by orange points.

**Figure S4**

**
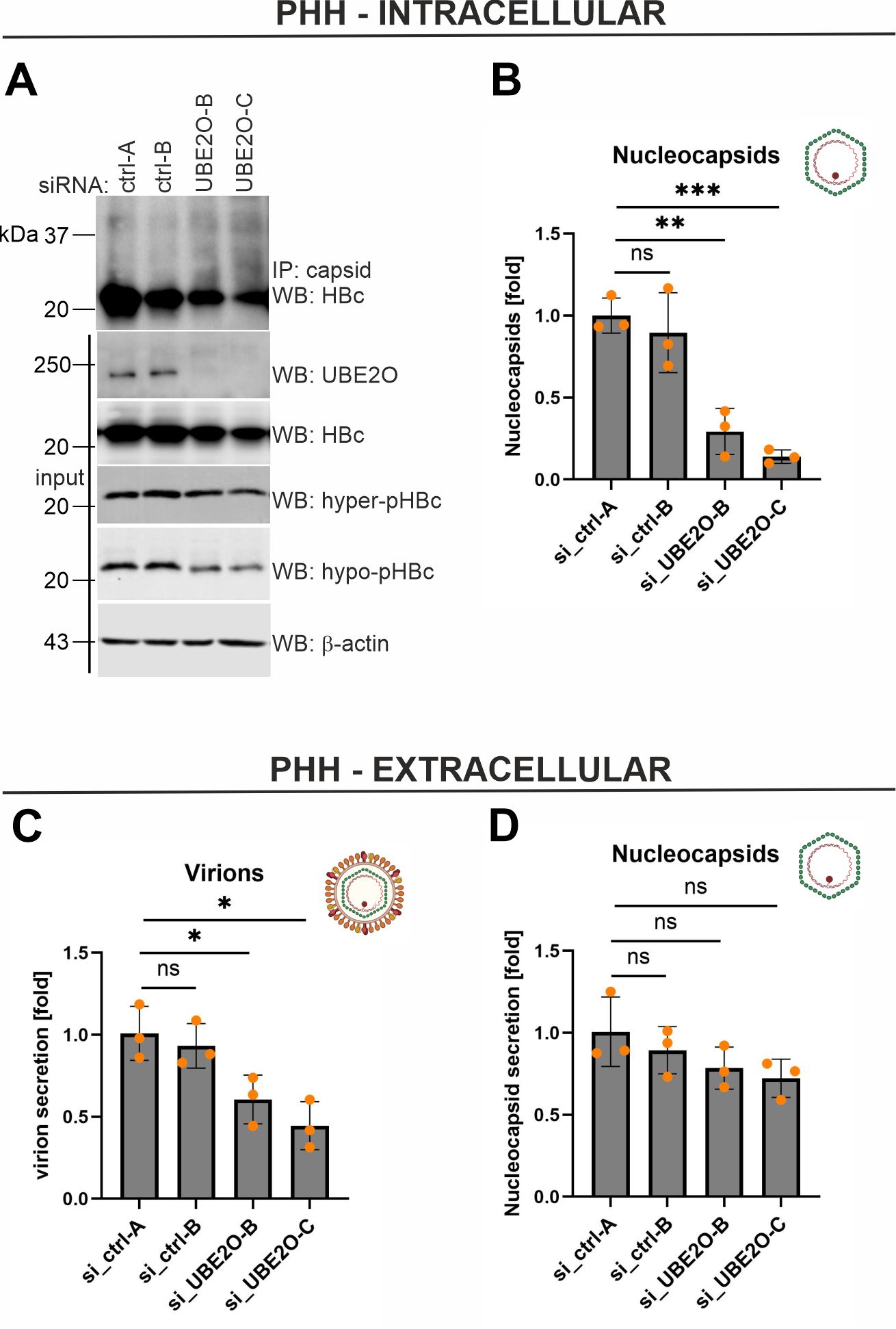
**

**Figure S4. UBE2O knockdown in HBV-infected primary human hepatocytes (PHH) led to the inhibition of intracellular nucleocapsid assembly and the secretion of enveloped virions.**

PHH were transfected with control and UBE2O-specific siRNAs. Two days post-transfection, the cells were infected with the HBV according to the experimental outline shown in Figure 2A. Six days post-infection, the cells and media were harvested for analyses of nucleocapsid assembly and virion secretion. **(A)** The protein extracts isolated from HBV-infected PHH were analyzed by Western blotting (WB) and subjected to immunoprecipitation with anti-capsid (Hyb-3120) antibodies (IP: capsid, WB: HBc). The expression levels of UBE2O, HBc, hyper-pHBc, and hypo-pHBc are shown for comparison (input). **(B)** Downregulation of UBE2O expression in HBV-infected PHH resulted in decreased levels of intracellular DNA-containing nucleocapsids. The protein extracts were immunoprecipitated with anti-capsid antibodies, and associated HBV DNA was quantified by qPCR. **(C)** Downregulation of UBE2O expression in HBV-infected PHH resulted in decreased levels of extracellular enveloped virions. The cell culture supernatants were immunoprecipitated with anti-S antibodies, and the associated HBV DNA was quantified via qPCR. **(D)** The naked nucleocapsid secretion was unaffected by UBE2O downregulation in HBV-infected PHH. Naked nucleocapsids were immunoprecipitated from cell culture supernatants using anti-capsid antibodies, and associated viral DNA was quantified via qPCR. In **(B-D)**, the data are presented as means ± SDs of one experiment performed in biological replicates (n = 3) shown by orange points. Statistical significance of differences between UBE2O silencing and control (si_ctrl-A) groups was evaluated by a two-tailed t-test (p ≥ 0.05 – not significant (ns); * p < 0.05; ** p < 0.01; *** p < 0.001).

**Figure S5**

**
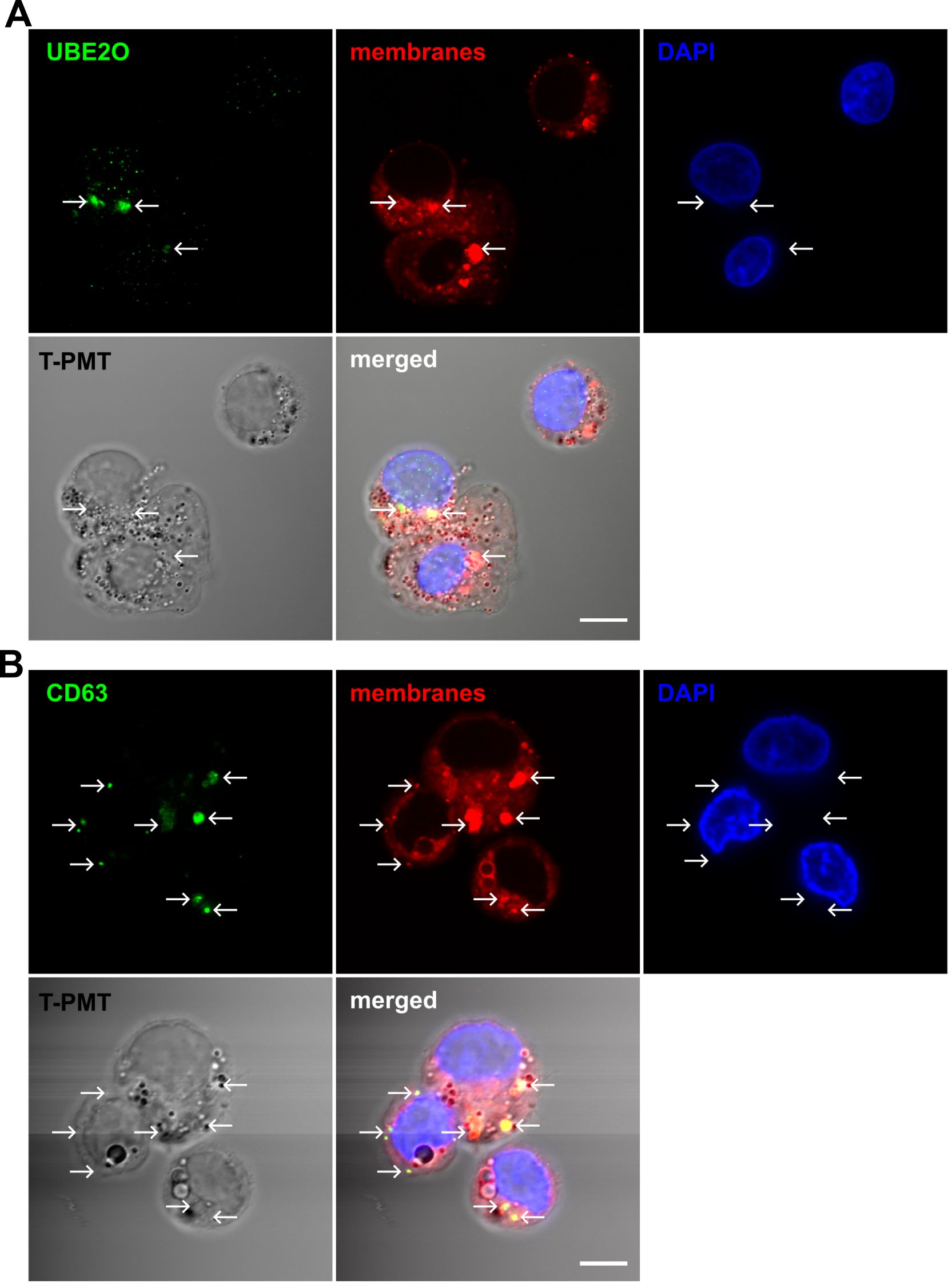
**

**Figure S5. Confocal sections of HepG2-NTCP cells.**

Cells growing on coverslips were fixed, permeabilized and stained with specific antibodies against UBE2O **(A)** or CD63 **(B)** (both shown in green). Cell membranes (red) were stained with the Abberior STAR ORANGE membrane probe. Cell nuclei (blue) were stained with DAPI. Transmitted light detector (T-PMT) was used to determine cell shape (grey). Arrows indicate overlapping regions between UBE2O/CD63 and membrane fluorescence signals. Bars, = 10 µm. Images taken with a Carl Zeiss LSM 880 NLO confocal microscope.

**Figure S6**

**
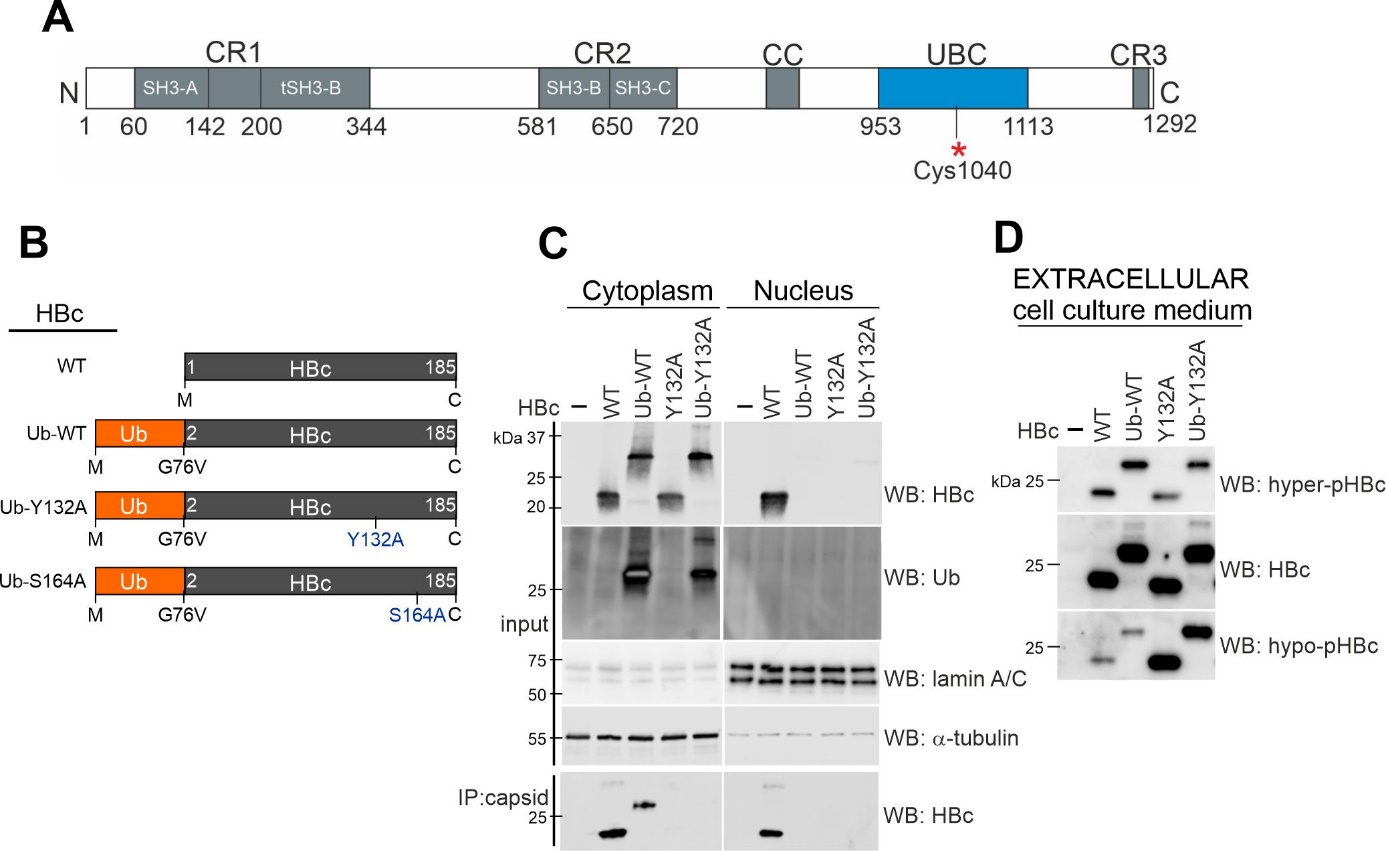
**

**Figure S6.** **UBE2O domain structure and analysis of intracellular localization, capsid assembly and secretion of HBc and Ub-fused HBc (Ub-HBc) variants.**

**(A)** Domain architecture of UBE2O. The UBE2O protein, composed of 1,292 amino acids, exhibits a complex domain architecture that includes three conserved regions (CR1, CR2, and CR3), a ubiquitin-conjugating (UBC) domain, and a predicted coiled-coil (CC) domain. It also features several SH3-like domains (SH3-A, SH3-B, and SH3-C) and tandem SH3-like domain (tSH3-B). The position of the catalytic cysteine residue (C1040), essential for ubiquitin transfer activity, is indicated by a red asterisk. Domain positions were obtained from UniProt ([www.uniprot.org](http://www.uniprot.org)). **(B)** Scheme of HBc and ubiquitin-fused HBc (Ub-HBc) variants. **(C, D)** HepG2-NTCP cells were transfected with HBc (wt, Y132A) and Ub-HBc fused variants (Ub-wt and Ub-Y132A). Forty-eight hours after transfection, the medium was collected and the cells were fractionated into cytoplasmic and nuclear extracts. **(C)** The cytoplasmic and nuclear protein lysates were analyzed by Western blotting with anti-HBc, anti-Ub, anti-lamin A/C (nuclear marker), and anti-α-tubulin (cytoplasmic marker) (input). Immunoprecipitation with anti-capsid antibodies (IP:capsid, WB:HBc) determined the ability of HBc variants to assemble into capsids. **(D)** The extracellular release of HBc and capsids was assessed in the culture medium by Western blotting using anti-HBc, anti-hyper-pHBc, and anti-hypo-pHBc antibodies.

**Figure S7**

**
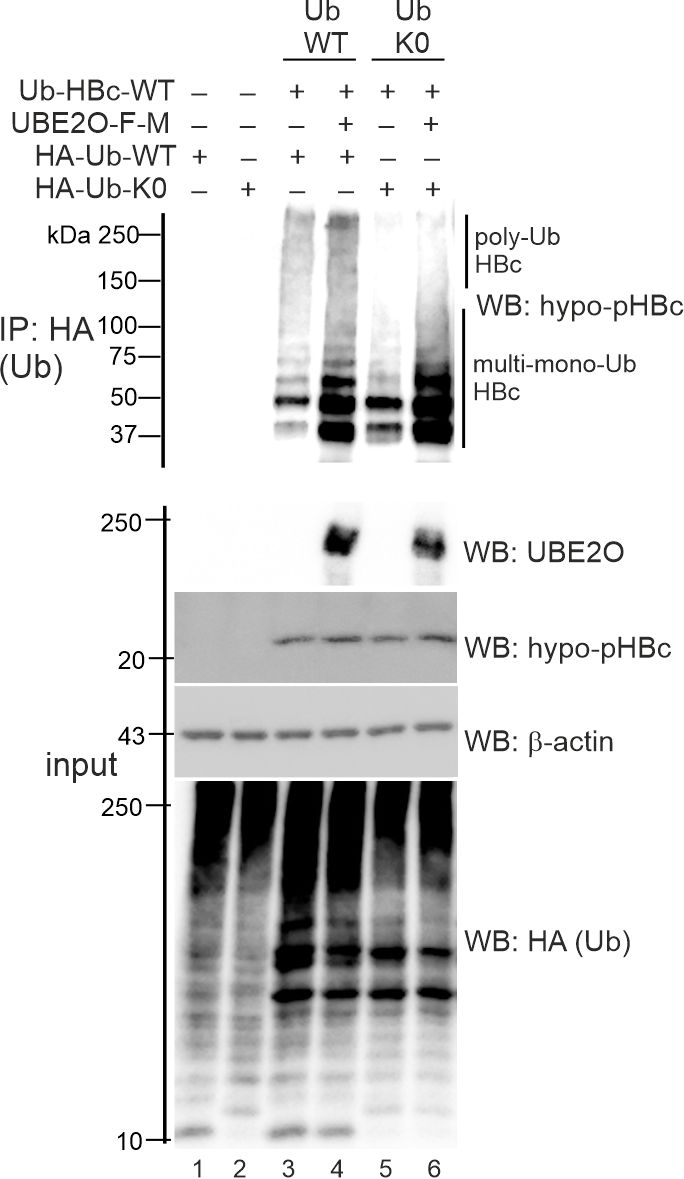
**

**Figure S7. Initial attachment of a single ubiquitin moiety to HBc promotes its subsequent multi-monoubiquitination by UBE2O.** Immunoprecipitation and Western blot analysis of cellular lysates from HepG2-NTCP cells transfected with Ub-HBc-wt, Flag-Myc-tagged wt-UBE2O and HA-tagged ubiquitin wt or K0 mutants. The ubiquitinated proteins were precipitated with anti-HA antibodies (IP: HA) and analyzed by Western blotting with anti-hypo-pHBc.

**Video S1. Z-stack imaging of UBE2O and CD63 using confocal microscopy.** HepG2-NTCP cells were fixed six days post-infection and immunostained with anti-HBc capsid (red) and anti-UBE2O (green) antibodies. Z-stack images of one individual cell were processed using ZEN blue software (ZEISS Microscopy, Germany).
